# Genome‐wide association study identifies quantitative trait loci associated with resistance to *Verticillium dahliae* race 3 in tomato

**DOI:** 10.1002/tpg2.70132

**Published:** 2025-10-14

**Authors:** Tika B. Adhikari, Bode A. Olukolu, Anju Pandey, Ashley N. Philbrick, Dilip R. Panthee, Reza Shekasteband, Randolph G. Gardner, Ralph A. Dean, Frank J. Louws

**Affiliations:** ^1^ Department of Entomology and Plant Pathology North Carolina State University Raleigh North Carolina USA; ^2^ Department of Entomology and Plant Pathology University of Tennessee Knoxville Tennessee USA; ^3^ Department of Horticultural Science North Carolina State University Raleigh North Carolina USA; ^4^ Mountain Horticultural Crops Research and Extension Center (MHCREC) Mills River North Carolina USA

## Abstract

Verticillium wilt (VW) disease, caused by *Verticillium dahliae* Kleb., is a major threat to tomato (*Solanum lycopersicum* L.) production. Identifying loci associated with VW resistance can accelerate breeding efforts and support sustainable disease management. Although the *Ve1* and *Ve2* genes confer resistance to *V. dahliae* races 1 and 2, the emergence of race 3 in the United States poses a new challenge. To investigate the genetic basis of quantitative resistance to the race 3 strain KJ14a, we evaluated 250 diverse tomato accessions. Disease severity and incidence were assessed weekly over 5 weeks, using chlorosis/necrosis percentage (CN_perc) and the number of symptomatic leaves (LC) as phenotypes. OmeSeq quantitative reduced‐representation sequencing yielded 42,941 high‐quality single nucleotide polymorphism and insertion‐deletion markers. Genome‐wide association study (GWAS) and local linkage disequilibrium analyses identified four candidate genes associated with VW resistance on chromosomes 3, 5, and 7, including two loci mapping to previously reported quantitative trait loci and two novel resistance loci on chromosome 5. The candidate genes are involved in plant defense and the modification of cell walls. To validate and assess the breeding potential of marker‐trait associations, we applied GWAS‐assisted best linear unbiased prediction (GWABLUP). Using an additive + dominance model and GWABLUP with top 100 associated markers, predictive ability for LC improved by 16.4% and 4.8%, and for CN_perc by 11.7% and 7.9%, compared to standard genomic best linear unbiased prediction using 100 and 18,000 genome‐wide markers, respectively. These results offer valuable insights into the genetic architecture of VW resistance to race 3 and demonstrate the potential of combining GWAS and genomic prediction to accelerate tomato breeding for durable disease resistance.

## Core Ideas

- Verticillium wilt (VW), caused by *Verticillium dahliae* Kleb., poses a significant threat to global tomato production.
- The extensive cultivation of tomato varieties containing the *Ve1* gene has led to an increase in the prevalence of race 2 and race 3 strains in tomato fields.
- Linkage disequilibrium (LD)‐based genome‐wide association study (GWAS) approach can identify genetic loci significantly associated with resistance to VW.
- Identify candidate genes linked to VW resistance and with predicted functions.
- A GWAS‐informed genomic prediction model can improve predictive ability and expedite the breeding process.

## Plain Language Summary

Verticillium wilt (VW) is a severe soilborne disease that poses a significant threat to tomato production. The phase‐out of fumigants like methyl bromide, along with the emergence of race 3 of *Verticillium dahliae* Kleb., underscores the need for sustainable resistance strategies. In this study, we employed genome‐wide association study (GWAS) analysis to identify four candidate genes associated with VW resistance in tomato, including two novel loci on chromosome 5. To evaluate their breeding potential, we applied a genomic prediction assisted by GWAS (GWABLUP). This approach improved predictive ability for VW resistance by up to 16.4% compared to standard genomic prediction models. Our findings provide new insights into the genetic basis of VW resistance, suggesting that the identified markers could be valuable tools for breeding more resilient tomato varieties.

**Abbreviations**
AM: association mapping
AUDPC: area under disease progress curve
FDR: false discovery rate
GBLUP: genomic best linear unbiased prediction
GBS: genotyping‐by‐sequencing
GP: genomic prediction
GRM: genetic relationship matrix
GS: genomic selection
GWABLUP: GWAS‐assisted best linear unbiased prediction
GWAS: genome‐wide association study
LD: linkage disequilibrium
MAF: minor allele frequency
NCSU: North Carolina State University
OmeSeq‐qRRS: OmeSeq quantitative reduced representation sequencing
PC: principal component
PCA: principal component analysis
QTL: quantitative trait loci
RIL: recombinant inbred line
RMIP: resample model inclusion probability
SNP: single nucleotide polymorphism
VW: Verticillium wilt

## 1 INTRODUCTION

Tomato (*Solanum lycopersicum* L.) is a globally important vegetable, with a 2021 global production of 186 million metric tons (FAOSTAT, 2023). Verticillium wilt (VW) is caused by the fungus *Verticillium dahliae* Kleb. and infects tomato roots, spreading through the vascular system and leading to V‐shaped chlorosis, which starts at the leaf tip and extends down the midrib (Berlanger & Powelson, 2000; Grogan et al., 1979; M. Ma et al., 2023; Wilhelm, 1955). This chlorosis eventually develops into necrotic lesions (Berlanger & Powelson, 2000). *Verticillium dahliae* produces microsclerotia that can remain viable for several years in soil (Wilhelm, 1955). Yield losses of up to 67% have been reported in susceptible tomato varieties due to VW (Grogan et al., 1979). Pre‐plant soil fumigants, such as methyl bromide, have effectively controlled VW in the temperate regions of the United States (Louws et al., 2006). However, the environmental impacts of methyl bromide have resulted in global bans (US EPA, 2018). Although grafting tomato varieties onto resistant rootstocks has effectively managed various soilborne pathogens, it is not commercially effective against *V. dahliae* (Louws et al., 2010). As a result, the focus has shifted toward identifying resistant tomato accessions and developing new cultivars suitable for fields infested with *V. dahliae (*Foolad, 2007).

A wild‐type cherry tomato (*S. lycopersicum* var. *cerasiforme)* has shown high resistance to *V. dahliae* (Schaible et al., 1951). This resistance is attributed to a single dominant gene, *Ve1*, which has been incorporated into modern commercial tomato cultivars (Schaible et al., 1951). Historically, the use of tomato cultivars that carry the *Ve1* gene has effectively managed VW (Jones & Grill, 1975). However, *V. dahliae* strains from tomatoes in California, along with a *Verticillium albo‐atrum* strain from potatoes in Canada, showed virulence against the *Ve1* resistance gene in the tomato cultivar Loran Blood (Dobinson et al., 1996). Several *V. dahliae* strains capable of overcoming *Ve1*‐mediated resistance have emerged in the United States. As a result, the *Ve1* gene was no longer effective against strains of race 2 in tomato in Ohio (Alexander, 1962), California (Grogan et al., 1979), and North Carolina (Bender & Shoemaker, 1977). In Japan, segregating F_2_ populations have been derived from rootstock cultivars, Aibou and Ganbarune‐Karis, and evaluated under controlled conditions (Usami et al., 2017). This resistance to race 2 is governed by a single dominant locus, *V2*, as demonstrated by the segregation of resistance in F_2_ populations derived from self‐fertilized rootstock cultivars (Usami et al., 2017). They reported that the current race 2 of *V. dahliae* should be divided into two categories: “race 2” (nonpathogenic on Aibou) and “race 3” (pathogenic on Aibou).

Our group has utilized these two rootstocks (Kano & Usami, 2019; Usami et al., 2017) along with whole‐genome sequencing of the representative strains of *V. dahliae* to identify race 3 in the temperate, mountainous tomato‐growing regions of North Carolina (Ingram et al., 2020). Among 10 wild accessions tested, two showed resistance to multiple pathogenic races of *V. dahliae* (Vermeulen et al., 2022). To investigate this resistance, recombinant inbred line (RIL) populations were created by crossing *Solanum pimpinellifolium* (G1.1596 population) and *Solanum cheesmanii* (G1.1615 population) with *S. lycopersicum* ‘Moneymaker’ (Vermeulen et al., 2022). Notably, no quantitative trait loci (QTLs) were identified in the G1.1596 population, whereas the G1.1615 population exhibited four small‐effect QTLs associated with reduced stunting (Vermeulen et al., 2022). The limited availability of genetic resources highlights the urgent need for further research into tomato disease resistance against *V. dahliae* race 3. Implementing a breeding strategy that focuses on genome‐wide association study (GWAS) to discover quantitative resistance to VW will be imperative for effective long‐term disease management (Acharya et al., 2020).

Recent advancements in genetic technologies have opened new opportunities for exploring disease resistance to *V. dahliae* in various crops such as alfalfa (L.‐X. Yu et al., 2017), cotton (Bolek et al., 2005; L. Wang et al., 2025; Y. Zhao et al., 2014, 2017), lettuce (Simko et al., 2022), oilseed rape (Rygulla et al., 2008), strawberry (Antanaviciute et al., 2015; Feldmann et al., 2023; Pincot et al., 2020), and tomato (Amanullah & Shekasteband, 2025; Vermeulen et al., 2022). Research often uncovers quantitative resistance governed by multiple QTLs, each contributing moderately to minor effects (Sikandar & Shekasteband, 2025). Innovative technologies, such as genotyping‐by‐sequencing (GBS) and GWAS, have advanced the identification of key markers linked to important agronomic traits (Beissinger et al., 2013; Elshire et al., 2011; Poland et al., 2012). GWAS, leveraging linkage disequilibrium (LD), is a powerful tool for revealing marker‐trait associations (MTAs) across diverse genetic materials (Tibbs Cortes et al., 2021). It relies on ancestral recombination events for precise mapping (Nordborg & Weigel, 2008). Understanding population structure shaped by geographic separation and selection is crucial in GWAS, as it can inflate false favorable rates (Porras‐Hurtado et al., 2013; Pritchard et al., 2000). Mixed linear models have been developed to effectively manage these challenges (Patterson et al., 2006; J. Yu et al., 2006). The goal of GWAS is to identify genetic markers and characterize trait architecture (Ding et al., 2015; Zhang et al., 2022), whereas genomic prediction (GP), known as genomic selection (GS), estimates breeding values for more efficient selection (Meuwissen et al., 2024). An emerging technique, GWAS‐assisted best linear unbiased prediction (GWABLUP), evaluates whether significant GWAS markers enhance prediction accuracy compared to traditional methods (Meuwissen et al., 2024; Zhang et al., 2022). We hypothesize that substantial allelic variation exists among tomato accessions, with specific markers associated with resistance to VW. This enables the identification of key genetic factors influencing resistance levels. Additionally, GP offers a promising approach for improving complex and polygenic traits, such as VW resistance, and the early prediction of resistance in breeding materials. This can enhance selection efficiency and accelerate the development of resistant tomato varieties.

**Core Ideas**

- Verticillium wilt (VW), caused by *Verticillium dahliae* Kleb., poses a significant threat to global tomato production.
- The extensive cultivation of tomato varieties containing the *Ve1* gene has led to an increase in the prevalence of race 2 and race 3 strains in tomato fields.
- Linkage disequilibrium (LD)‐based genome‐wide association study (GWAS) approach can identify genetic loci significantly associated with resistance to VW.
- Identify candidate genes linked to VW resistance and with predicted functions.
- A GWAS‐informed genomic prediction model can improve predictive ability and expedite the breeding process.

The widespread adoption of tomato varieties with the *Ve‐1* gene has led to an increase in race 2 and race 3 strains in tomato fields in the United States, thereby overcoming the resistance that was previously effective against race 1 (Alexander, 1962; Hall et al., 1972). This situation underscores the importance of ongoing research to identify new sources of resistance, particularly in developing strategies against evolving and highly virulent strains, such as race 3 (Ingram et al., 2020). Although obtaining precise estimates of yield losses caused by race 3 is challenging, its importance is widely recognized. Even a conservative estimate of a 5% reduction in yield due to the disease in North Carolina alone could lead to millions of dollars in losses. This disease can severely damage crops, resulting in both reduced yield and compromised quality. Additionally, it is essential to mention that VW is a soilborne pathogen capable of persisting in the soil for extended periods (Subbarao et al., 1997; Wilhelm, 1955), making its management challenging once a field becomes infected. In this study, we assessed 250 tomato landraces, wild relatives, and advanced breeding lines for resistance to *V. dahliae* race 3 strain KJ14a (Ingram et al., 2020). The objectives were to (i) identify genetic loci and candidate genes significantly associated with resistance to *V. dahliae* race 3 using LD‐based GWAS, and (ii) confirm GWAS results by demonstrating that top GWAS hits can provide greater genomic predictive ability (PA) than genome‐wide markers. These findings provide valuable insights into the development of tomato cultivars resistant to *V. dahliae* race 3 strain KJ14a.

## 2 MATERIALS AND METHODS

### 2.1 Tomato accessions

A panel of 250 diverse tomato lines comprise wild relatives, landraces, advanced breeding lines (hereafter referred to as GWAS panel) were requested from various sources (File S1) including (i) the Plant Genetic Resources Unit, USDA, ARS, NEA in Geneva, NY ([Plant Introduction [PI], *n* = 172]), which was kindly provided by Sherri L Tennies, (ii) the Tomato Genetics Resource Center in the Department of Plant Sciences at the University of California, Davis, CA ([*Lycopersicon* accession [LA], *n* = 15]), which Maxine Nixon kindly supplied, and (iii) the tomato breeding programs (*n* = 53) at North Carolina State University (NCSU) led by Drs. Randolph Gardner, Dilip Panthee, and Reza Shekasteband, NCSU, MHCREC, Mills River, NC, and other sources (*n* = 10). In this study, the “tomato accession” herein refers to a seed sample that distinctly identifies a cultivar, breeding line, landrace, or population.

### 2.2 Greenhouse planting conditions

Tomato seeds of different accessions were planted directly in a Ray Leach cone‐tainer single stubby cell (3.8 cm diameter and 22 cm long) system and placed in an RL98 tray (Stuewe and Sons, In., Corvallis, OR). These trays were filled with Sun Grow potting soil, which consists of Canadian sphagnum peat moss (70%–80%), vermiculite (5%–10%), and dolomite limestone. The seedlings were watered daily and fertilized weekly with “Miracle‐Gro Water‐Soluble All‐Purpose Plant Food” at a rate of 1 g per 1000 mL. The plants were exposed to a photosynthetic photon flux density of 251 µmoL photon m^−2^ s^−1^, with a blue, green, and red percentage of 37%, 37.1%, and 25.9%, respectively, for 12 h each day. The temperature was maintained between 24°C and 28°C, and the relative humidity ranged between 30% and 50%.

### 2.3 Inoculum preparation and inoculation

*Verticillium dahliae* race 3 strain KJ14a (Ingram et al., 2020) was taken from −80°C and grown on potato dextrose agar (PDA: 39 g L^−1^, streptomycin 50 mg L^−1^, Difco Lab.). The culture plates were incubated at 28°C for 10 days. Conidia were harvested from the PDA plates, washed, and filtered through a double layer of autoclaved cheesecloth. They were then diluted to a final concentration of 5 × 10^6^ conidia mL^−1^ using a hemacytometer. Three‐week‐old tomato seedlings were inoculated by drenching them with 5 mL of inoculum near the base of the plant stem using a 10‐mL sterile pipette.

### 2.4 Disease assessment

After inoculation, yellow blotches developed on lower leaves, and necrotic V‐shaped lesions appeared at the tips of older leaves, which are distinctive symptoms of VW (Conover, 1959). To assess root discoloration or vascular colonization, destructive sampling was required, which was unfeasible because we needed to monitor foliar disease progression over time. Therefore, disease incidence was assessed by visually counting the number of symptomatic leaves (LC) with V‐shaped lesions on each plant. The disease severity (%) in terms of chlorosis and necrosis percentage (CN_perc) symptoms in each plant was rated on a scale from 0 to 5, where 0 indicates no symptoms, 1 = <25% chlorotic or necrotic leaves, 2 = 25%–50% chlorotic or necrotic on lower two leaves, 3 = 50%–75% chlorotic or necrotic on two to three leaves, 4 = more than 75% chlorotic or necrotic on more than three leaves, and 5 = 100% or complete necrosis. The disease progression was monitored daily for 5 weeks, starting 1 week after inoculation. In the susceptible check, “Bonny Best,” chlorosis and necrosis symptoms were visible 6 days after inoculation. The values for the area under the disease progress curve (AUDPC) for AUDPC_LC and AUDPC_CN_perc were computed (Shaner & Finney, 1977) based on the disease incidence and severity scores, respectively.

### 2.5 Experimental design and phenotypic data analysis

We conducted two experiments in the greenhouse of the Department of Entomology and Plant Pathology at NCSU, Raleigh, NC. To minimize variations in plant growth, inoculum load, pathogen progression, and environmental conditions, these experiments were carried out simultaneously in two different locations (benches) within the greenhouse. Each plant grown in a pot was considered a replication, with each plant serving as an experimental unit. In total, there were six plants per accession in each experiment, resulting in 12 plants/accession across both experiments (Table S1). Each experiment was conducted using a randomized complete block design with six replications. Replications were considered a random effect, while tomato accessions were regarded as a fixed effect. The scored phenotypic data were transformed using the square root method to ensure normality and to correct for variance heterogeneity. The phenotypic data from 250 accessions for each experiment were subjected to analysis of variance (ANOVA) using R statistical software version 4.3.1 (R Core Team, 2023). This analysis aimed to determine the interaction between accession (G) and environment (E) in the disease scoring of tomatoes affected by VW. Since we found no significant genotype–environment (G × E) interactions (*p* < 0.05), we combined the phenotypic data from both experiments. These combined phenotypic data from both experiments were used to compute the best linear unbiased estimates (BLUEs) and used for GWAS and GPs.

The coefficient of determination (*R* ^2^), regression parameters (slope and intercept), their standard errors, and the relationship between the disease assessment variables (AUDPC_LC and AUDPC_CN_perc) were calculated (R Core Team, 2023). Accessions with an AUDPC_LC value of less than 20 and an AUDPC_CN_perc value of less than 50 were classified as resistant. On the other hand, accessions with an AUDPC_LC value exceeding 20 and an AUDPC_CN_perc value of more than 50 were considered susceptible. The least significant difference was used to compare the AUDPC_LC and AUDPC_CN_perc values when the effects were statistically significant at *p* ≤ 0.001.

### 2.6 Genomic DNA extraction

Young leaf tissues were collected from 3‐week‐old seedlings grown in the greenhouse at the Department of Entomology and Plant Pathology, NCSU, Raleigh, NC. The collected leaf tissues were lyophilized and ground to a fine powder in 2‐mL tubes with a quarter‐inch ceramic bead using a tissue homogenizer (Model D1030‐E, Beadbug, Benchmark Scientific Inc.) at 400 rpm for 2 min. DNA was extracted from 100 mg of each accession using the DNeasy Plant Mini kit (Qiagen) according to the manufacturer's instructions. The DNA concentration was quantified using a NanoDrop spectrophotometer ND‐1000 (NanoDrop Technologies Inc.). Approximately 20 ng of DNA sample was digested with the Eco*RI* restriction enzyme (New England Biolabs) at 37°C for 2 h. The quality of the digested DNA was evaluated at 1% agarose by gel electrophoresis. After verifying the quality, the purified DNA accessions from the GWAS panel, normalized to a concentration of 100 ng µL^−1^, were transferred into 96‐well skirted plates. Three plates were shipped to BASEwise Solutions LLC, for OmeSeq‐qRRRS library preparation and next‐generation sequencing.

### 2.7 OmeSeq‐qRRS library preparation and sequencing

An OmeSeq quantitative reduced representation sequencing (OmeSeq‐qRRS) method was performed as described previously (Kuster et al., 2021; Rico et al., 2022). To prepare libraries, purified DNA was quantified using the Quant‐iT PicoGreen dsDNA Assay Kit (Invitrogen Corporation) and normalized to 20 ng µL^−1^. The DNA accessions were subjected to DNA nick repair. DNA libraries were prepared by a two‐step sequential double‐digestion of genomic DNA with *NsiI‐HF* and *NlaIII* (New England Biolabs) while incorporating custom ssDNA Illumina P5 and P7 adapters (96 × 96 variable length barcodes/UMI), respectively, after each digest. The genomic DNA fragments were incorporated into custom P5 and P7 adapters using the strand displacement activity and isothermal amplification of Bst 2.0 WarmStart DNA polymerase (New England Biolabs, Inc.). The annealing was limited to the 4‐bp overhangs while maintaining the double‐stranded state of the template DNA, thereby preventing off‐target annealing of adapters. The variable‐length barcodes (7–10 bp) enable multiplexing of up to 9216 accessions and were preceded by 6‐bp buffer sequences on the adapters. This was done to ensure the barcodes were in a region of the NGS (next‐generation sequencing) reads with high‐quality base calls. Paired‐end sequencing (2 × 150) was performed on one lane using the Illumina NovaSeq × Plus platform (Illumina Inc.).

### 2.8 Demultiplexing and quality filtering

The sequenced library was demultiplexed and quality‐filtered using ngsComposer (Kuster et al., 2021). For quality filtering, the ngsComposer parameters included trimming off the 6‐bp buffer sequence before the barcode sequences, demultiplexing with no more than 1‐bp mismatch, removal of reads with sequencing error in restriction enzyme motifs, and individual reads were trimmed based on an end quality score threshold of 20 within a 10‐base sliding window. After trimming, the minimum read length was set at 64, and adapter removal was performed at a minimum of 12 base matches. Finally, a minimum Phred (CodonCode Corp.) quality score of 20 was required for at least 80% of the bases in each read.

### 2.9 Variant calling and filtering

Using the quality‐filtered Fastq files, variant filtering and variant calling were performed using the GBSapp automated pipeline (Bararyenya et al., 2020; Brůna et al., 2023; Rico et al., 2022), which uses both original and third‐party tools (NextGenMap, samtools, bcftools, bedtools, and picard), and GATK Ver. 4.2.6.1 Tools for variant calling and filtering (Lin et al., 2014; Su et al., 2021; https://solgenomics.net/organism/Solanum_lycopersicum/genome). The genotypic scores of 0, 1, and 2 indicate the number of alternate alleles and represent homozygous for the reference allele (0), heterozygous (1), and homozygous for the alternate allele (2), respectively. The parameters for variant filtering included a minor allele frequency (MAF) threshold of 0.02 and a minimum read depth of 6 for each accession call. For filtering missing data, variants, and accessions dropped iteratively with no accession calls across each sample. The two missing rate thresholds were applied since sensitivity to missing data varies for different genetic analyses. The 30% missing rate threshold across accessions minimizes missing data (42,941 markers and 235 accessions) for the GWAS, GP (with an additional MAF threshold of 0.05, resulting in 18,503 markers), and LD analysis. In comparison, the 5% missing rate threshold across accessions further minimizes missing data (8578 markers and 226 accessions) for population differentiation analyses (i.e., STRUCTURE analysis, phylogenetic analysis, and principal component analysis [PCA]), as well as additional GWAS to compare models.

Imputation of missing accession data was performed using the mode imputation method, which assigns the most frequent accession at each marker to missing entries (Mora‐Márquez et al., 2024). This approach is efficient and straightforward for datasets with low missingness; however, it can bias results toward the major allele, especially when rare alleles, such as resistance variants, are present. To reduce this risk, we limited missing data to 20% per marker and performed imputation to ensure sufficient minor allele representation (MAF > 0.02). More advanced methods (e.g., Beagle, k‐NN, and random forest) may better capture rare variants but require phased data or dense reference panels (e.g., parents in bi‐parental populations), which are not feasible for this genetically diverse panel. We interpreted results involving low‐frequency alleles with appropriate caution. Specifically, for analyses that integrated marker and phenotypic data, such as GWAS and GP, only accessions present in both datasets were included.

### 2.10 Population structure and phylogenetic analysis

Knowledge of the population structure is vital for GWAS to avoid spurious associations (Patterson et al., 2006). We carried out 10 independent runs for each value of the assumed subpopulations (*K*), ranging from 1 to 6 in the STRUCTURE Ver. 2.3.4 software (Pritchard et al., 2000). The delta‐*K* plot was used to determine the optimal value of *K* (Earl & vonHoldt, 2012). For each *K*, the burn‐in period was set to 5000 iterations, and the number of Markov chain Monte Carlo repetitions after burn‐in was set to 50,000 iterations. PCA was performed using the additive relatedness matrix, computed based on the van Raden method, in the AGHmatrix R package (Amadeu et al., 2016). Using the first two principal components (PCs), PCA plots were generated with the FactoMineR and factoextra R packages (Husson et al., 2014; Kassambara & Mundt, 2020). The neighbor‐joining‐based phylogenetic analysis was conducted with 1000 bootstraps and implemented in the DARwin software version. 6.0.021 (Perrier et al., 2003).

### 2.11 LD analysis

We analyzed GWAS and local LD using the 42,941 filtered marker dataset across 235 tomato accessions. To quantify the extent of genome‐wide and local LD decay around each significantly associated marker, we computed LD estimates (*R* ^2^) using the genetics R package and visualized the region under consideration using the ggplot2 R package (R Core Team, 2018; Wickham, 2016). The genome‐wide LD was represented as scatter plots and box plots, which show LD decay on a chromosome basis. Marker pair LD estimates were plotted based on the map distances of the tomato reference genome SL4.0, corresponding to *S. lycopersicum* cv. Heinz 1706 (Lin et al., 2014; Su et al., 2021; https://solgenomics.net/organism/Solanum_lycopersicum/genome).

### 2.12 Genome‐wide association study

In total, 42,941 high‐quality markers across 235 tomato accessions were used to determine associations between markers and phenotypes (i.e., AUDPC_LC and AUDPC_CN_perc values). The GWAS analysis was performed using a linear mixed model and implemented in the R package GWASpoly. The VanRaden method (VanRaden, 2008) was employed to calculate the kinship (*K*) matrix, which was subsequently modeled as a random effect to account for spurious associations resulting from population structure. Manhattan plots were used to visualize the distribution of marker *p*‐values across all the tomato chromosomes, with the marker positions plotted on the *X*‐axis and −log (*p* value) plotted on the *Y*‐axis. The quantile–quantile (*Q*–*Q*) plots were used to evaluate false discovery rates (FDRs) and detection power.

### 2.13 MTAs and gene annotations

We used the Bonferroni correction and FDR (Benjamini & Hochberg, 1995) threshold to determine the significance of the MTA. The Bonferroni correction threshold at 5.93 was computed based on the formula *α*/*n*, where *α* = 0.05 and *n* = total number of markers. Markers above the threshold lines are considered significant. The percentage of variance explained by a marker (*R* ^2^) was estimated based on modification of a previously described method (Rickman et al., 2025):

$$\begin{array}{ccc} \mathrm{PV}E_{\mathrm{additive}} & = & \frac{\mathrm{Var}\left(\mathrm{SNP}\right)}{\mathrm{Total}\;\mathrm{phenotypic}\;\mathrm{variance}\;\left(\sigma^{2}\right)}\\ & = & \frac{2p_{j}\left(1-p_{j}\right)\beta_{j}^{2}}{\sigma^{2}} \end{array}$$

where 2 is the ploidy level for the diploid genome (genotype coded as 0, 1, 2), $p_{j}$ is the allele frequency (reference allele) of the marker in the population, 1 − $p_{j}$ is the frequency of the alternative allele, *β_j_* represents the effect size of the marker j (from GWAS results), and $\sigma^{2}$ is the total phenotypic variance of the trait.

Genetic loci significantly associated with resistance to *V. dahliae* race 3 strain KJ14a were annotated using the tomato reference genome version. SL4.0 IS derived from the *S. lycopersicum* cv. Heinz 1706 (Su et al., 2021; https://solgenomics.net/organism/Solanum_lycopersicum/genome). To verify the predicted gene models in the tomato reference genome, we used the BLAST tool with the core nucleotide database. The Conserved Domain search was conducted on the National Center for Biotechnology Information website (Marchler‐Bauer et al., 2005). We employed specific approaches to identify and prioritize candidate genes within the QTL regions. First, the candidate genes identified through GWAS were found to be significantly associated with the markers. Second, these candidate genes were assessed for their location within coding regions or 100 kb of associated markers. Third, we performed LD analysis to determine whether each candidate gene was in LD with the associated marker, using an LD threshold of 𝑟^2^ ≥ 0.2 (LD‐based GWAS approach). Finally, the putative functions of candidate genes linked to MTAs were inferred based on BLAST searches, conserved domain analysis, and functional characterization of orthologs reported in the literature.

### 2.14 Genomic prediction

Two GP models, genomic best linear unbiased prediction (GBLUP) and GWABLUP, were compared to assess whether the top GWAS hits for the kinship matrix (genetic relationship matrix, GRM) are sufficient to produce similar predictions and higher GP accuracy than genome‐wide markers (Daetwyler et al., 2013). This will validate that top hits are biologically relevant and are enriched with actual positive hits. The GBLUP model utilized 18,503 markers (filtered from 42,941 based on MAF ≥ 0.05) and 100 randomly selected markers, while the GWABLUP model used the top 100 GWAS hits to compute the GRM. For GWABLUP, (i) the GWAS was performed on the training dataset for each independent iteration instead of the entire population; (ii) 300 markers with high effect estimates were initially selected; (iii) markers with zero variance were excluded, and one of two correlated markers (Spearman correlation ≥ 0.8) was removed to avoid multicollinearity; and (iv) the mlmm.gwas R package (Segura et al., 2012) was used to identify the top 100 GWAS hits based on model selection. The model selection involved calculating the resample model inclusion probability (RMIP) using fivefold cross‐validation and 100 iterations. After each iteration of model selection, 100 markers with the highest effects were selected. After 100 iterations, 100 markers with the highest RMIP values (inclusion frequency of the predictor variable) were selected and used for computing the GRM. Unlike the approach used by Meuwissen et al. (2024), our implementation of GWABLUP does not involve constructing a weighted genomic relationship matrix.

GP was performed using the GAPIT R package version 3.0 (Lipka et al., 2012), applying a fivefold cross‐validation method with 100 iterations for each model. Predictive abilities were compared across six models, including comparisons between GBLUP and GWABLUP, as well as among the three dosage effect models. The GBLUP model is described as y=1nμ+Zu+ε, where *y* represents the vector of phenotypes (number of phenotypes × 1), 1*n* is the vector of ones, *μ* is the overall mean, *α* is a c‐vector of specified covariates (fixed effects) corresponding to coefficients including the intercept, *Z* is the known design matrix for accessions, and *u* is the random effects. The model assumes $u\;∼\;N(0,\sigma_{a}^{2}Kor\sigma_{a+d+ad}^{2}K)$ with *K*  =  kinship matrix with *a* (additive), *d* (dominance), or *ad* (additive + dominance); and that $\;\varepsilon \;∼\;N(0,\sigma_{e}^{2}I)$. The GWABLUP model is similar to GBLUP, except that it utilizes the top 100 GWAS hits to compute the GRM, rather than using genome‐wide marker data. The AGHmatrix R package (Amadeu et al., 2016) was used to calculate additive and dominance GRM, as well as a matrix that accounts for both additive and nonadditive effects using VanRaden and Vitezica methods (VanRaden, 2008; Vitezica et al., 2013). The PA was computed by performing a Pearson correlation analysis between observed BLUEs and the genome‐estimated breeding values (GEBVs). The results for each trait across the models were visualized using box plots. Model performance was compared using a one‐way ANOVA, and the results were also visualized in the box plots.

## 3 RESULTS

### 3.1 Phenotypic evaluation

**FIGURE 1:**
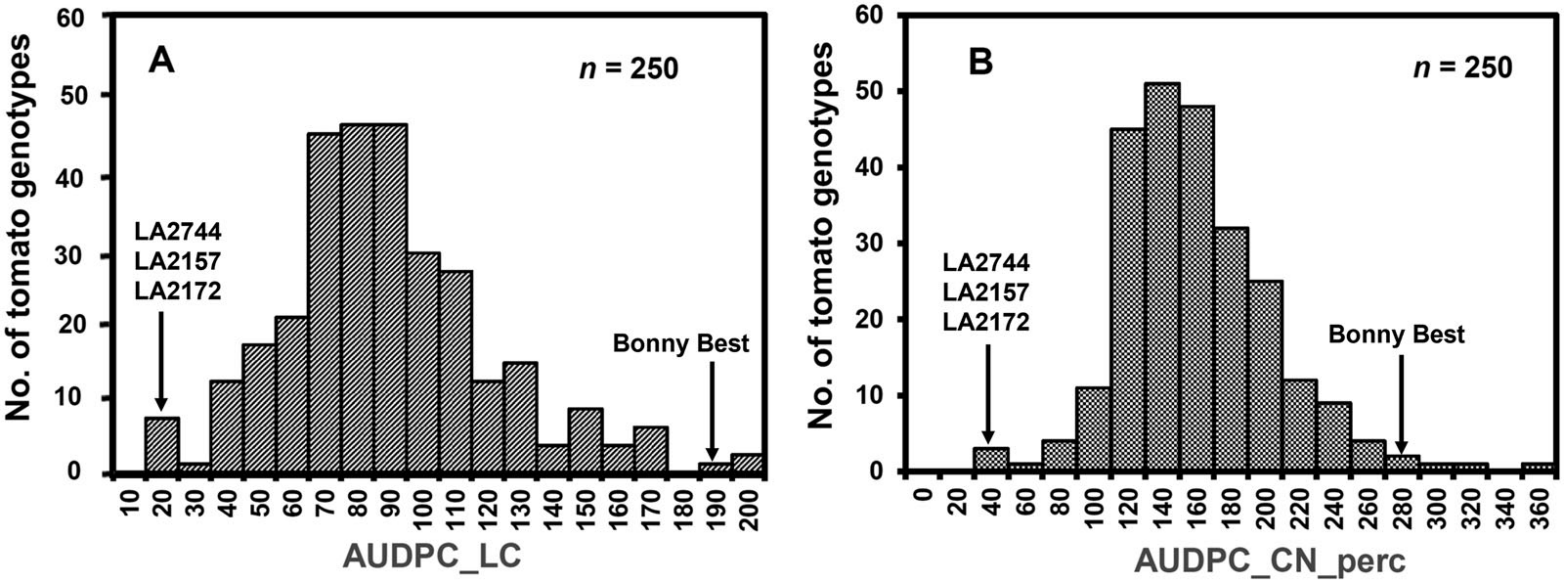
Histogram shows the distribution of phenotypic data across 250 tomato accessions inoculated with *Verticillium dahliae* race 3 strain KJ14a. Over 5 weeks, the plants were evaluated weekly for disease incidence (LC) in panel (A) and severity (CN) in panel (B), which were used to calculate the area under the disease progress curve (AUDPC). Accessions with AUDPC_LC below 20 and AUDPC_CN below 50 were considered resistant. The cultivar Bonny Best was used as a susceptible control.

**FIGURE 2:**
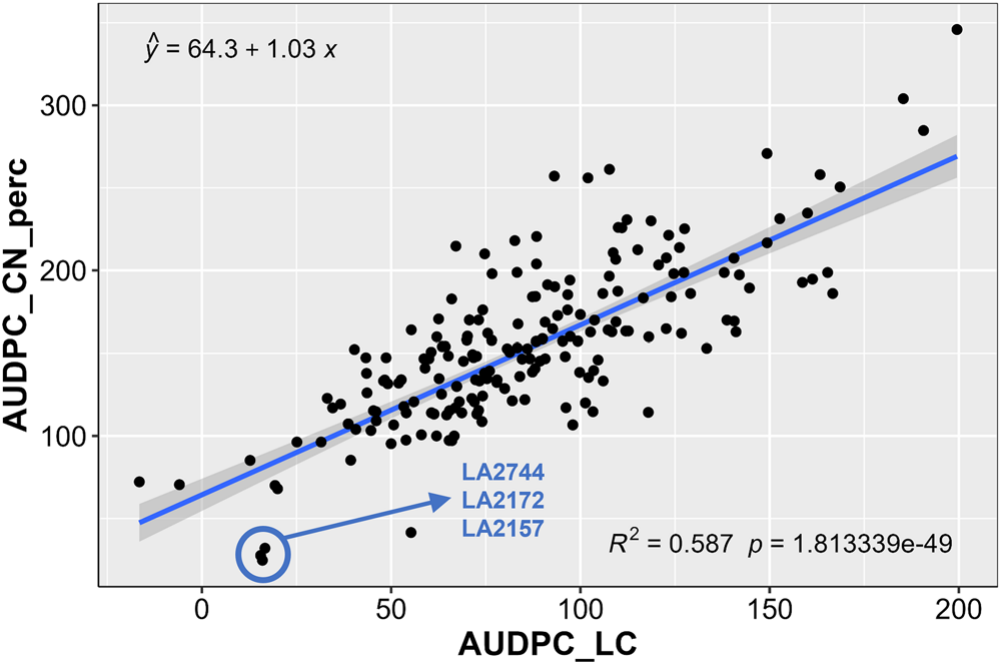
A regression analysis was conducted on a panel of 250 tomato accessions inoculated with *Verticillium dahliae* race 3 strain KJ14a. The plants were monitored over a 5‐week period, with weekly assessments of disease incidence (LC) scores and disease severity percentages (CN_perc). These ratings were from two experiments and then used to calculate the area under the disease progress curve (AUDPC). The AUDPC_LC and AUDPC_CN_perc values were square root‐transformed for analysis, and three of the most resistant lines (crop wild relatives) are shown in blue text.

The results of AUDPC_LC and AUDPC_CN_perc showed substantial phenotypic variability for VW resistance in the GWAS panel (Figure 1A,B). The AUDPC_LC and AUDPC_CN_perc values had a normal distribution. The relationship between AUDPC_LC and AUDPC_CN_perc was linear and was significantly high at 0.77 (Figure 2). The coefficient of variation was significant (*R* ^2 ^= 0.59; *p* < 0.001). The tomato accessions were classified as resistant based on their AUDPC_LC value of less than 20 and AUDPC_CN_perc value of less than 50. The AUDPC_LC values for resistant accessions ranged from 12 to 20, while the AUDPC_CN_perc values for resistant accessions ranged from 19 to 42 and were significantly different (*p* ≤ 0.001) from susceptible ones (Table 1). Among the 250 tomato accessions evaluated, 97.5% were susceptible or moderately resistant to *V. dahliae* race 3. Three wild relative tomato accessions, *Solanum peruvianum* LA2744 from northern Chile to central Peru and *Solanum arcanum* LA2157 and LA2172 from Peru, were highly resistant, indicating the presence of potential sources of resistance to *V. dahliae* race 3.

**TABLE 1:** Verticillium wilt disease ratings for seven of the 250 most resistant tomato accessions. Ratings are based on the mean area under the disease progress curve (AUDPC) for disease incidence and severity following drench inoculation with *Verticillium dahliae* race 3 strain KJ14a.

Genotype	AUDPC_LC^a^	AUDPC_CN_perc^b^
NC13192_F5	13a	85a–g
LA2744	16a	25ab
LA2172	16a	28a–c
LA2157	17ab	32a–d
LA1708	19a‐c	70a–f
LA1361	20a–c	68a–f
PI270435	55a–f	41a–e
Bonny Best^c^	191ef	285g–i

*Note*: Mean values with the same letters were not statistically significant (*p* ≤ 0.001).

^a^ AUDPC_LC: number of leaves with V‐shaped lesions (LC), with values ≤20.

^b^ AUPDC_CN_perc: percentage of leaf chlorosis and necrosis with values ≤50.

^c^ Bonny Best was used as a susceptible check.

### 3.2 Variant calling and genotyping

**FIGURE 3:**
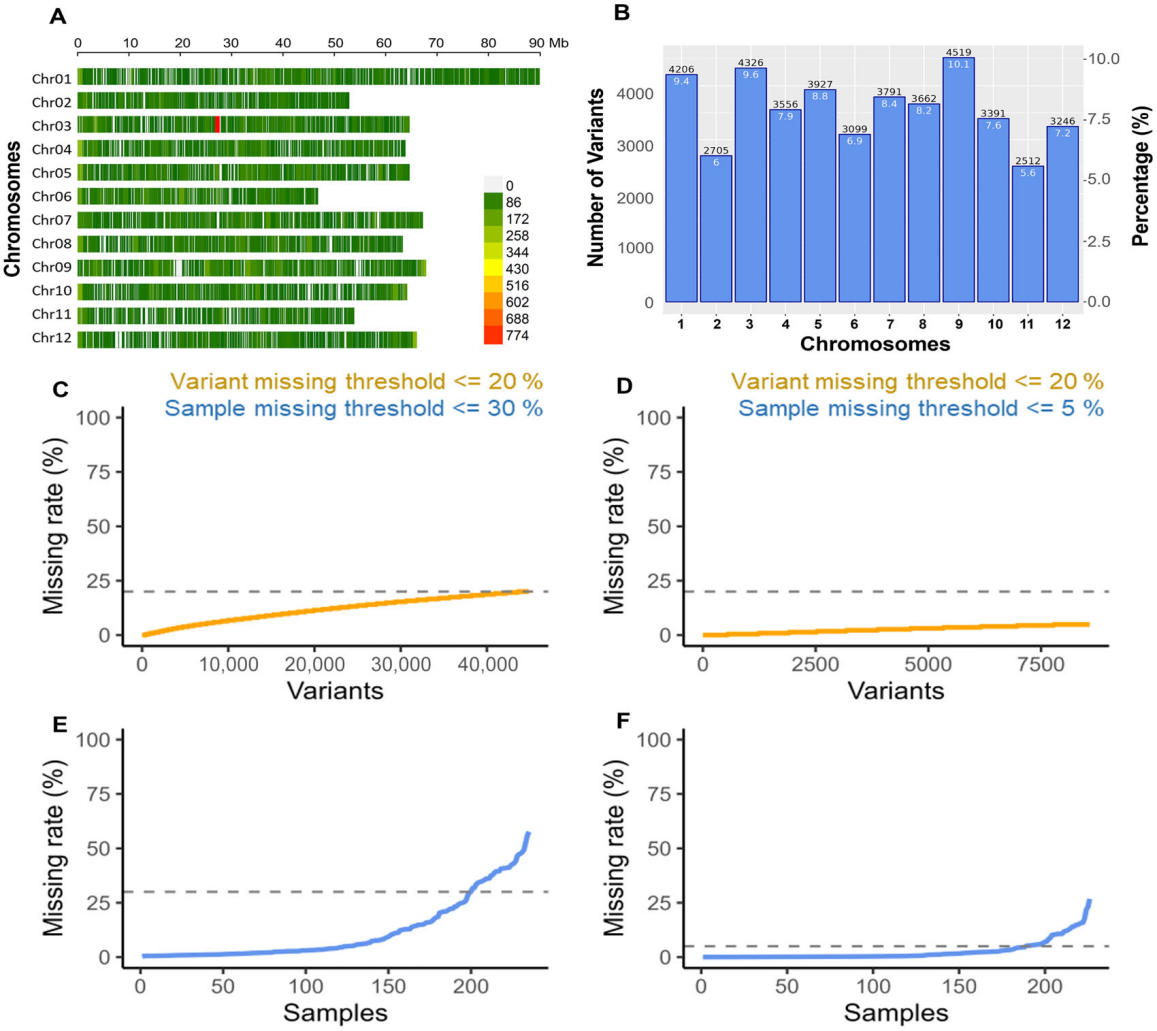
The analysis presents the chromosome‐wise marker density and distribution of 42,941 single nucleotide polymorphism (SNP) and insertion‐deletion (INDEL) markers (A and B). These markers were derived from a diverse panel consisting of 235 tomato accessions. Panel A displays a bar plot illustrating the marker density across the physical reference genome. In Panel B, the bar plot shows the number (above bar, primary axis) and percentage (inside bar, secondary axis) of markers per chromosome. Panels C and F feature line graphs that depict the missing rate thresholds that yield 42,941 and 8578 markers with 235 and 226 accessions, respectively. The yellow and blue color trend lines indicate variant and accession missing rates, respectively, for the 42,941 (panels C and E) and 8578 (panels D and F) marker datasets.

An OmeSeq‐qRRS library based on 250 tomato accessions was sequenced and utilized for variant calling using the GBSapp automated pipeline. The read depth distribution across the accessions was visualized with a boxplot (Figure S1). The homozygous accession calls accounted for 89% of the marker data (87% and 2% homozygous for reference and alternate allele, respectively), while the heterozygous accession calls accounted for 11% (Figure S2). The mean and median read depth distribution (following unbiased down‐sampling) are 23 and 26, respectively, across the tomato diversity population (Figure S3). The median and mean MAF are 0.04 and 0.07, respectively (Figure S4). Based on an average marker density interval of 18.6 kb (total of 42,941 markers) and an average gene density of 25.7 kb per gene (35,000 protein‐coding genes over 858 Mb genome), the distribution of variants on a chromosome‐by‐chromosome basis revealed gene‐level resolution in the tomato genome (Figure 3A). The markers spanned the entire length of the tomato chromosomes with a size that varied from 45.5 Mb for chromosome 6 to 91 Mb for chromosome 1. The marker density varied across the 12 genome chromosomes, with the highest number of markers (4519 markers; 10.1%) found in chromosome 9 and the lowest (2512 markers, 5.6%) in chromosome 11 (Figure 3B). The missingness rate was visualized with a line graph (Figure 3C–F) and a heat map (Figure S5).

### 3.3 Population structure analysis

The delta (Δ)*K* method enabled us to determine the total number of subpopulations by evaluating population structure with K ranging from 1 to 10. Based on the membership and Delta K methods, the panel of tomato accessions contained four subpopulations (Figure S6A,B). A phylogenetic analysis based on neighbor‐joining (1000 bootstraps) and a PCA was performed to validate the STRUCTURE result. With high bootstrap values, the phylogenetic tree confirmed the membership and clustering of the four distinct subpopulations (Figure S6C), which were inferred from the STRUCTURE analysis. The PCA result also confirmed four subpopulations, with the first (44.8%) and second (13.6%) PCs accounting for 58.4% of the genetic variation (Figure S7). The high cos2 values revealed that the accessions within each of the four subpopulations have high statistical confidence for clustering together. The most resistant accessions used in this analysis were obtained from the University of California, Davis, CA (Figures 1 and 2) and clustered within the admixed group (Figure S7). Subpopulation 1 is predominantly comprised of NCSU breeding lines. Subpopulation 2 consists of only a few lines, comprising USDA PI accessions and LA lines. Subpopulation 3 is predominantly comprised of USDA PI accessions. Additional subpopulations (*K* = 5–7) were evaluated, but only four distinct subpopulations were consistently resolved (Figures S8 and S9). As *K* increased, the number of individuals in the admixed group slightly increased, while the composition of the four subpopulations remained essentially unchanged.

### 3.4 MTAs and candidate gene annotations

**FIGURE 4:**
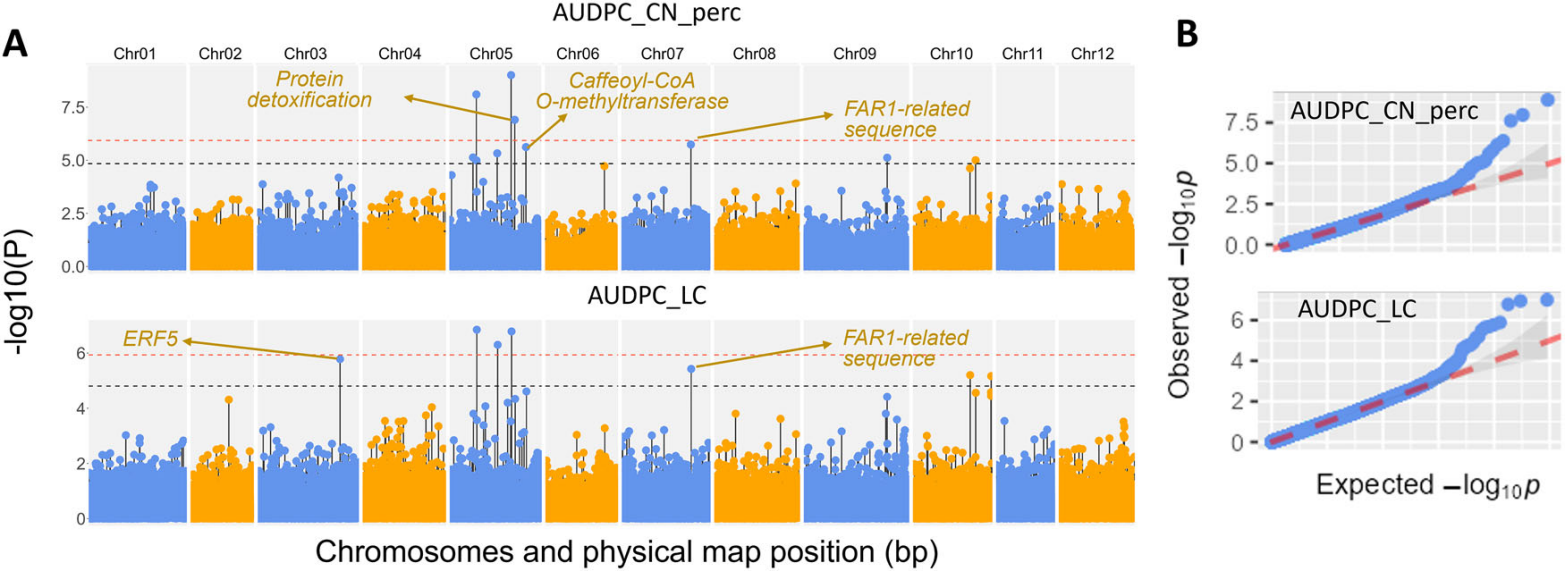
The Manhattan plot (panel A) illustrates genome‐wide associations related to resistance against *Verticillium dahliae *race 3 strain KJ14a, using 42,941 markers across 205 tomato accessions. The *Y*‐axis displays the −log10 of *p*‐values, while the *X*‐axis shows the chromosomal positions. The red and gray dashed lines represent the Bonferroni correction and the false discovery rate (FDR) significance thresholds, respectively. In panel B, the quantile–quantile (*Q–Q*) plots compare observed versus expected *p*‐values, revealing a low FDR and high detection power.

Based on the AUDPC_LC and AUDPC_CN_perc phenotypic data, four candidate genes were identified as being linked to MTAs on chromosomes 3, 5, and 7, conferring resistance to *V. dahliae* race 3. While many more MTAs passed significance thresholds (Figure 4A), only these four candidates could be confirmed following local LD analysis. The *Q–Q* plots showed a low FDR and strong detection power (Figure 4B). Two of the candidate genes, located on chromosome 5, are novel (Figure 4A), as there are no previous reports of resistance QTL on this chromosome. While the associated locus on chromosome 7 was located between two metabolically linked genes, FAR‐RED impaired response 1 (FAR1) and FAR1‐related sequence 1 (FRS1) at 6.6–69.4 kb (Table 2), only FRS1 was in LD with the associated marker. The other candidate genes include several important defense‐related genes (Table 2): ethylene‐responsive transcription factor 5 (ERF5), protein detoxification (DTX), and caffeoyl‐CoA O‐methyltransferase (CCoAOMT). Each of the associated markers accounted for 8.2%–16.1% of the phenotypic variation (*R* ^2^). A positive effect estimate indicates that the alternate allele contributes to increased resistance compared to the reference allele (Table 2). Lines with homozygote accessions (reference allele) for the associated markers showed greater resistance than those homozygous for the alternate allele (Table 3). The heterozygotes tended to moderate resistance, suggesting that superior alleles confer partial dominance for resistance. This finding aligns with the results from the GP analysis (below), where the additive + dominance model yielded better PA than using additive or dominance GRM alone.

**TABLE 2:** List of candidate genes located near significantly associated single nucleotide polymorphisms (SNPs) underlying variation in resistance to Verticillium wilt. Only genes in linkage disequilibrium (LD) with associated markers were designated as candidate genes. Predicted gene functions are also provided.

Marker ID	*R* ^2^ ^a^ (%)	Gene position^b^(bp)	Score^c^	Ref^d^	Alt^e^	MAF	Effect	Distance (kb)^f^	Gene ortholog^g^
**AUDPC_CN_perc (area under the disease progression curve based on V‐shaped lesions on leaves)**									
Chr05_44454259	16.1	Chr05:44470756–44470962	6.91	T	G	0.02	64.6	16.5	Protein DETOXIFICATION^1^
Chr05_52351676	14.8	Ch05:52315718–52316061	5.62	T	C	0.06	35.1	35.6	Caffeoyl‐CoA O‐methyltransferase^1,2^
Chr07_52845993	13.2	Chr07:52881843–52882164	5.74	A	G	0.04	50.7	69.4	FAR1‐related sequence^3^
**AUDPC_LC (area under the disease progression curve based on count of symptomatic leaves)**									
Chr03_49371879	11.2	Chr03:49461880–49462566	5.78	T	C	0.09	26.2	15.4	Ethylene‐responsive transcription factor 5^5^
Chr07_52845993	8.2	Chr07:52881843–52882164	5.43	A	G	0.04	38.4	69.4	FAR1‐related sequence^3^

Abbreviation: MAF, minor allele frequency.

^a^ *R* ^2^ (%): Percentage of phenotypic variation explained.

^b^ Gene start and stop position.

^c^ Score: −log_10_ of *p*‐value.

^d^ Reference allele.

^e^ Alternate allele.

^f^ Proximity of SNP position to the beginning or end of the candidate gene.

^g^ Putative functions of candidate genes: ^1^plant defense response, ^2^cell wall modification, ^3^light‐mediated defense response, ^4^reactive oxygen species (ROS) production, and ^5^ethylene signaling.

**TABLE 3:** Genotype calls of significantly associated markers and corresponding ranges of disease scores. Homozygotes for the reference allele tend to be the most resistant, while homozygotes for the alternate allele are generally the most susceptible. Heterozygotes typically exhibit intermediate resistance.

Associated marker		Hom‐Ref allele	Het	Hom‐Alternate allele
Chr05_44454259	Accession	**TT**	**TG**	**GG**
	LC range	0–191	65–153	185–200
	CN_perc range	28–285	97–231	304–346
Chr05_52351676	Accession	**TT**	**TC**	**CC**
	LC range	0–191	61–153	84–200
	CN_perc range	28–285	114–231	147–346
Chr07_52845993	Accession	**AA**	**AG**	–^a^
	LC range	0–165	61–200	–
	CN_perc range	28–256	113–346	–
Chr03_49371879	Accession	**TT**	**TC**	**CC**
	LC range	0–200	51–161	80–185
	CN_perc range	71–346	97–230	129–304

^a^ The absence of homozygotes for the alternate allele at position 52,849,993 bp on chromosome 7 suggests that the alternate allele may be deleterious.

Furthermore, GWAS was conducted using 8578 markers to evaluate the impact of different models: (i) kinship alone (mixed linear model [MLM] with kinship as a random effect), (ii) STRUCTURE alone (GLM with STRUCTURE as a fixed effect), and (iii) both kinship and STRUCTURE (MLM with STRUCTURE as a fixed effect and kinship as a random effect). For models incorporating STRUCTURE, subpopulation sizes ranging from four to seven were tested. The GWAS result based on the 8578‐marker dataset (with a lower missing rate) (Figure S10) showed reduced detection power due to the lower marker resolution compared to the 42,941‐marker dataset. Based on the *Q–Q* plot, using STRUCTURE alone resulted in the highest false positive rates across all subpopulations (*K* = 4–7, Figure S11A,D). Combining STRUCTURE and the kinship matrix reduced detection power (Figure S11A,D), indicating potential over‐correction of population stratification, which increases false negatives. In contrast, using the kinship matrix alone consistently yielded the lowest false positive rate and the highest power of detection.

### 3.5 LD analysis

**FIGURE 5:**
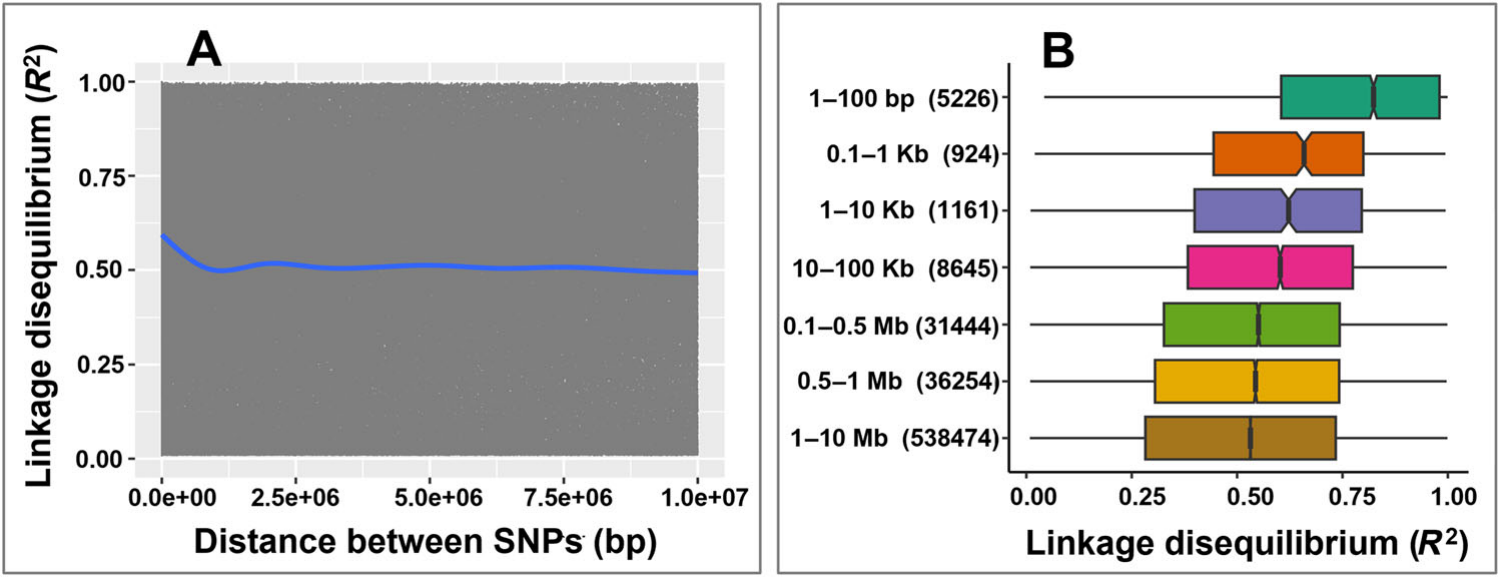
Genome‐wide linkage disequilibrium (LD) plot (panel A) and boxplot showing the genome‐wide distribution of LD estimates across genomic intervals (panel B). LD analysis was performed using 42,941 markers across 235 tomato accessions. SNP, single nucleotide polymorphism.

Associated markers identified through GWAS can be located within the coding sequence, the promoter region of genes, or in proximity to a gene. For the latter, evaluating local LD between the associated marker and the candidate gene is important for establishing that the candidate gene might be linked to resistance (Figure S12A,B). As expected, there was high LD between markers across the genome, and LD decayed slowly with the increasing physical distance between markers (Figure 5A,B). This means a marker associated with a trait might be linked to multiple genes within the same large LD block. For gene‐poor regions associated with markers surrounded by large noncoding sequences or genomic areas showing rapid LD decay, a local LD analysis can more accurately select candidate genes. The selected candidate genes identified in this study exhibit this pattern. Local LD analysis around the associated markers revealed both broad (97, 199, and 285 kb) and narrow (9 kb) LD blocks (Figure S12A). This confirms that the extent of LD decay varies throughout the genome (Figure 5A,B). Although some genes were identified within the 100 kb window (close to the average LD decay of 865.7 kb), LD between the candidate genes and the associated genes was not established. Consequently, these genes were not declared as candidate genes (Figure S12B).

### 3.6 GWAS‐assisted GP

**FIGURE 6:**
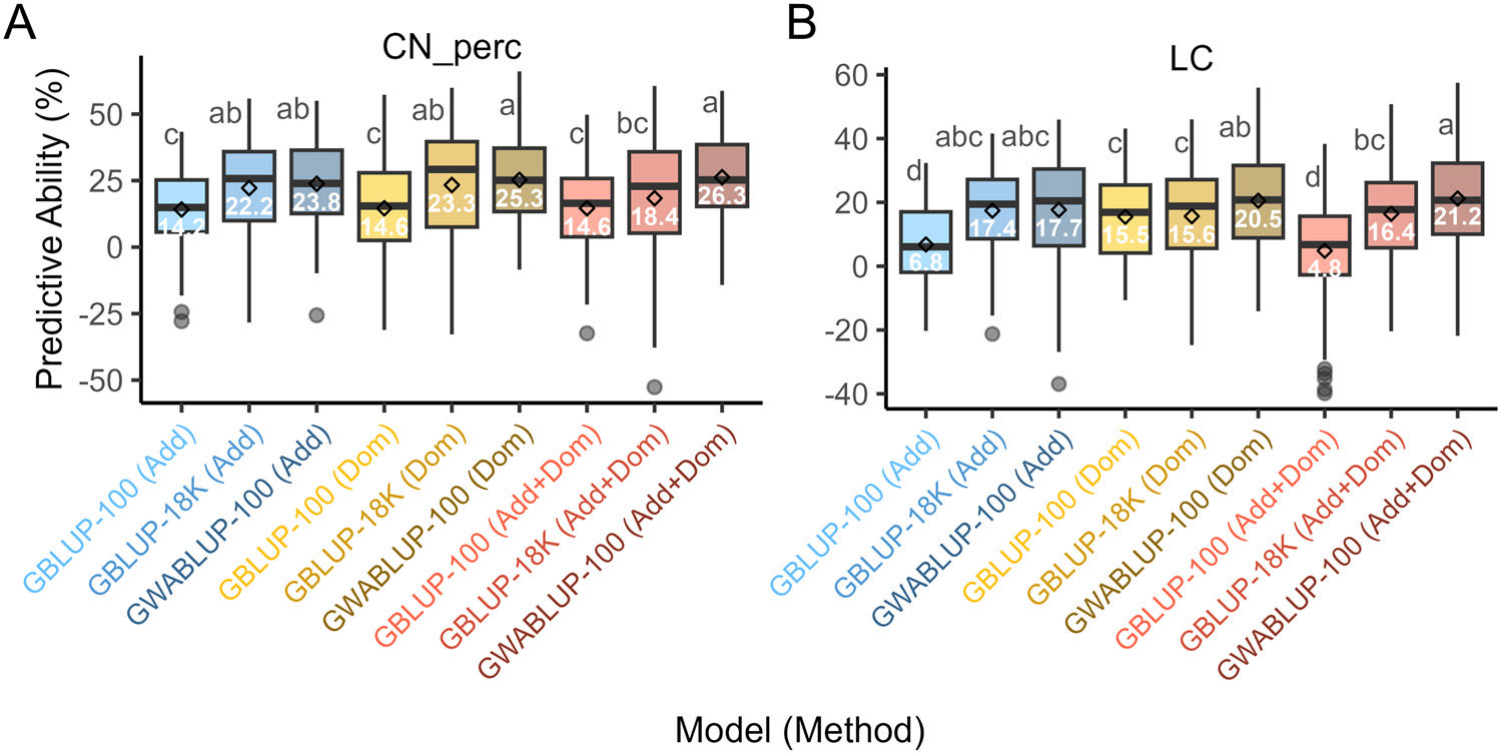
The genomic predictive abilities (PA) for LC (A) and CN_perc (B) were computed using genomic best linear unbiased prediction (GBLUP) and GWAS‐assisted best linear unbiased prediction (GWABLUP). This analysis modeled additive (Add), dominance (Dom), and both additive and dominance (Add + Dom) effects. For marker selection, the top 100 genome‐wide association study (GWAS) hits were identified from each of the 100 iterations of GWAS and model selection, and the final top 100 GWAS hits were based on markers with the highest resample model inclusion probability (RMIP) values. In the box plots, the white text inside the boxes indicates the mean PA values, while the letters from the Duncan multiple range test (DMRT) show the significance of differences between the means. The GBLUP model is based on 100 randomly selected and 18,503 genome‐wide markers.

GWABLUP improved PA for both LC and CN_perc traits (Figure 6A,B). The GWABLUP‐100 model, which utilizes 100 most associated markers, showed a significant enhancement in PA compared to the GBLUP‐100 model across all effect models for both traits. However, when comparing GWABLUP‐100 to the GBLUP‐18K model, which is based on a genome‐wide set of 18,503 markers, the improvement in PA was significant only for the Add + Dom model (the most predictive model) in LC and CN_perc, and for the Dom model in LC. There was no significant difference between GWABLUP‐100 and GBLUP‐18K for the Add model in LC and CN_perc and the Dom model in CN_perc (Figure 6A,B). Specifically, GWABLUP‐100 increased PA by 16.4% for LC and 11.7% for CN_perc in the Add + Dom model when compared to GBLUP‐100. The gains varied across effect models, ranging from 5% to 16.4% for LC and from 9.6% to 10.7% for CN_perc. In comparison to GBLUP‐18K, GWABLUP‐100 enhanced PA by 4.8% for LC and 7.9% for CN_perc, with improvements ranging from 0.3% to 4.9% for LC and from 2% to 7.9% for CN_perc across different effect models (Figure 6A,B).

## 4 DISCUSSION

*Verticillium dahliae* race 3, recently detected in North Carolina, poses a growing threat to tomato production. Resistance to races 1 and 2 is controlled by single genes such as *Ve1* and *V2* (Kawchuk et al., 2001; Usmani et al., 2017). However, these genes are ineffective against *V. dahliae* race 3, which seems to be governed by multiple genes and exhibits quantitative resistance (Vermeulen et al., 2022). We conducted an LD‐based GWAS on diverse tomato accessions and identified four candidate genes located on chromosomes 3, 5, and 7. These four candidate genes identified are involved in defense signaling, detoxification, and cell wall modification, mechanisms that align with known resistance pathways against plant pathogens. This supports the hypothesis that resistance to *V. dahliae* race 3 strain KJ14a may depend on multiple minor‐effect loci instead of classic *R*‐genes. By integrating GWAS and GWABLUP models, we improved PA by up to 16% for symptomatic leaf count and 12% for chlorosis severity, demonstrating a straightforward approach for incorporating markers into breeding pipelines. This approach, which utilizes selected markers to enhance GP compared to random genome‐wide markers, has been successfully deployed in other studies (Rickman et al., 2025; Z. Wang et al., 2025).

Although wild relatives lack favorable horticultural traits, they offer great potential as the primary gene pool for resistance to various plant pathogens (Khazaei & Madduri, 2022). In previous studies, the wild tomato species *Solanum habrochaites* accession LA407 was found to be resistant to several plant pathogens, including *Phytophthora capsici* (Quesada‐Ocampo & Hausbeck, 2010), as well as bacterial canker caused by *Clavibacter michiganensis* subsp. *michiganensis* (Kabelka et al., 2002), and early blight caused by *Alternaria solani* (Foolad et al., 2008). Our findings were consistent with previous studies on *V. dahliae* (Ingram et al., 2020; Yadeta, 2012). Among the evaluated accessions, three wild relatives (*S. peruvianum* accession LA2744, *S. arcanum* accession LA2157, and *S. arcanum* accession LA2172) exhibited high resistance to *V. dahliae* race 3 strain KJ14a. Interestingly, accession LA2744, which is resistant to *V. dahliae* race 3, also exhibits broad resistance to tomato spotted wilt virus strains (Kumar & Irulappan, 1992; Maluf et al., 1991; Paterson et al., 1989; Stevens et al., 1994). LA2157 provides heat‐stable resistance to *Meloidogyne incognita* (Jiang et al., 2023). These three highly resistant accessions identified in this study are being backcrossed with an elite tomato breeding line of *S. lycopersicum* to produce progeny that are resistant to the pathogen. This will also facilitate fine mapping of resistance gene(s) and investigation of interactions between *V. dahliae* and tomato.

Understanding population structure is essential for minimizing false positives in GWAS and ensuring accurate interpretations of genetic effects and population dynamics during breeding (Brachi et al., 2011). In this study, we identified three major subpopulations and one admixed group. Our findings from PCA clusters, STRUCTURE subpopulations, and neighbor‐joining phylogenetic tree clades all supported one another. Using the STRUCTURE software, we determined the optimal number of subpopulations by analyzing the delta‐*K* plot, which allowed us to evaluate the potential number of subpopulations ranging from 1 to 10. Preserving the genetic resources of wild relatives is crucial for meeting future crop demands, and effective management of these resources necessitates detailed information about the origins of accessions and their genetic diversity, which can be obtained through molecular techniques (Kim et al., 2017).

LD helps identify genetic markers associated with QTLs and provides insights into the evolutionary history and population structure (Jorde, 2000). The strength of LD and genome‐wide LD can be influenced by various factors such as domestication events (Caicedo et al., 2007), breeding practices (McNally et al., 2009), selection processes (Palaisa et al., 2004), outcrossing (McNally et al., 2009), and admixture (Lexer et al., 2007). In this study, we evaluated the extent of LD on a genome‐wide scale in wild relatives and admixture and discovered extensive LD blocks; specifically, LD tended to decay over large genomic regions of up to 865.7 kb in *Solanum lycopersicum* accessions, as expected for an inbreeding species (X. Liu et al., 2017). Notably, LD‐based GWAS has identified QTLs and markers associated with VW resistance in tomato. These findings can expedite the development of molecular markers and can be effectively used for breeding tomatoes resistant to VW, ultimately saving time and resources compared to traditional breeding methods.

RIL mapping populations generated from *S. pimpinellifolium* (G1.1596) and *S. cheesmanii* (G1.1615) were developed and evaluated for QTLs associated with resistance to the *V. dahliae* race 3 strain DVDS29, which is characterized by reduced stunting (Vermeulen et al., 2022). However, no QTLs were detected in the G1.1596 RIL population. The effects of the QTLs from *S. cheesmanii* G1.1615 on chromosomes 1 and 7 were observed, showing that the stunting in G1.1615 was lower compared to the combined QTLs from the other populations (Vermeulen et al., 2022). Although a larger set of tomato accessions shows promise, major genes for resistance were not identified, nor were strong QTL effects observed. This indicates that major resistance genes for *V. dahliae* are rare, possibly due to the pathogen's latent and endophytic lifestyle in natural environments (Vermeulen et al., 2022). Previous studies on other crops affected by *V. dahliae*, such as cotton (Bolek et al., 2005; G. Wang et al., 2008; Y. Zhao et al., 2014), eggplant (Toppino et al., 2016), oilseed rape (Rygulla et al., 2008), lettuce (Simko et al., 2022), and strawberries (Antanaviciute et al., 2015), also did not identify major resistance genes. In this study, GWAS and LD analyses identified four candidate genes linked to VW resistance on chromosomes 3, 5, and 7. This includes two loci corresponding to previously identified QTLs (Vermeulen et al., 2022) and two novel resistance loci found on chromosome 5. Two new resistance loci were identified on chromosome 5. The discovery of these loci presents new opportunities for understanding and enhancing resistance to VW. These findings significantly improve our understanding of the genetic basis of resistance to *V. dahliae* race 3 strain KJ14a and provide potential strategies for breeding disease‐resistant tomatoes.

One of the associated loci implicated the protein detoxification gene, which is a detoxification efflux carrier (DTX)/multidrug and toxic compound extrusion transporter. DTX genes have been shown to play a role in the establishment of plant disease resistance (Dobritzsch et al., 2016; Sun et al., 2011) and in response to other abiotic stresses (Amin et al., 2024). Another candidate gene, ERF5, is a critical component of the plant defense response pathway and interacts with several proteins, including ERF6, ERF8, and two chitin‐activated mitogen‐activated protein kinases (MPK3 and MPK6) (Son et al., 2012). It was found that ERF5 negatively regulates chitin signaling and plant defense against *Alternaria brassicicola* but positively regulates salicylic acid (SA) signaling and plant innate immunity against *Pseudomonas syringae* pv. *tomato* (Son et al., 2012). ERF5 genes play a crucial role in plant defense by regulating defense pathways in response to plant pathogens (Berrocal‐Lobo et al., 2002). Further research is necessary to understand the physiological roles of specific ERF genes in response to *V. dahliae*.

The candidate gene, identified on chromosome 7 as FRS1, was first discovered in *Arabidopsis thaliana* (L. Ma & Li, 2018). It plays a crucial role in regulating the balance between light and hormone‐signaling pathways that assist plants in their growth and defense responses (Y. Liu et al., 2019). A previous study found that the FHY3 and FAR1 genes negatively impact SA accumulation and resistance to the biotrophic bacterial pathogen *Pseudomonas syringae* pv. *tomato* (W. Wang et al., 2016). In addition, *fhy3 far1* double mutants were found to have high levels of SA and reactive oxygen species (ROS), which can trigger constitutive defense responses (W. Wang et al., 2016). The CCoAOMT candidate gene, found on tomato chromosome 5, plays a vital role in the phenylpropanoid pathway and lignin biosynthesis (M. Liu et al., 2023). Previous studies have shown that resistant chickpea plants exhibit increased expression of CCoAOMT in response to Fusarium wilt, resulting in higher lignification compared to susceptible plants (Meshram et al., 2019). In the case of VW, the upregulation of lignin biosynthesis pathways has been associated with resistance in various crops such as *Brassica* (Obermeier et al., 2013), cotton (Li et al., 2019), and wheat (Funnell‐Harris et al., 2024). CCoAOMT has been directly implicated in ROS signaling cascades following the hypersensitive response and has been shown to form a complex with an *R*‐gene to modulate the defense response. By enhancing the production of ferulate and sinapate esters, CCoAOMT contributes to oxidative cross‐linking, strengthening structural barriers (Olukolu et al., 2014; G. F. Wang & Balint‐Kurti, 2016). Additionally, in tomatoes, it has been observed that CCoAOMT enhances lignin biosynthesis and improves resistance to VW (Q. Zhao & Dixon, 2014). Thus, the CCoAOMT gene appears to be valuable for investigating phenylpropanoid‐based resistance to VW in tomatoes.

GP revolutionizes the breeding process by utilizing genomic information to predict individual performance for specific traits, thereby accelerating selection without the need for extensive phenotyping (Budhlakoti et al., 2022; Crossa et al., 2016). Advancements in affordable, high‐throughput genotyping technologies enable breeding programs to gather comprehensive and reliable genomic data, improving selection decisions through approaches such as GWAS (Crossa et al., 2016; Norman et al., 2018). Previous studies have demonstrated that the prediction accuracy of GS models can be enhanced through several methods, including increasing training population size, evaluating models on genetically similar test populations, implementing different GS algorithms, increasing marker density, and integrating significantly associated markers as fixed effects (Solberg et al., 2008; Norman et al., 2018). The current study assessed the predictive accuracy of GP models that integrate GWAS markers as fixed effects and explored the genetic architecture of disease resistance to VW by analyzing both additive and dominance effects. We computed both additive and dominance genetic relationship matrices (GRMs) and determined PA. In addition, we created a matrix that accounts for both additive and dominance effects (VanRaden, 2008; Vitezica et al., 2013). This approach effectively controlled the false‐positive rates stemming from the complex population structure and kinship present in our tomato panel. Consequently, we successfully identified markers that are significantly associated with both additive and dominance effects. By focusing on the top 100 markers identified from GWAS, we obtained superior genomic estimates compared to using a broader set of 18,503 markers. Moreover, filtering single nucleotide polymorphisms based on their associations in the GWAS improved the accuracy of GP for the VW resistance we investigated.

Our comparison of GP models revealed that the GWABLUP model enhanced PA for LC and CN_perc traits. The GWABLUP‐100 model, using 100 markers, showed substantial gains compared to the GBLUP‐100 model, particularly in the additive plus dominance model. Specifically, GWABLUP‐100 improved predictive accuracy by 16.4% for LC and 11.7% for CN_perc over GBLUP‐100, and by 4.8% for LC and 7.9% for CN_perc compared to GBLUP‐18K. These results indicate that using markers from GWAS improves GP accuracy, consistent with findings in previous studies that combined GWAS and GP in maize (Bian & Holland, 2017), rice (Spindel et al., 2016), and winter wheat (Herter et al., 2019). This research, along with previous studies (Daetwyler et al., 2014; Crossa et al., 2016), demonstrates that GP can provide GEBVs for QTLs associated with the trait of interest. Additionally, quantitative resistance to the *V. dahliae* pathogen in strawberry was significantly improved using training data from elite and exotic hybrids, achieving GP accuracies of 0.47–0.48, which potentially explained 70%–75% of the additive genetic variance for resistance to VW (Feldmann et al., 2023). In the current study, our models focusing on dominance effects performed similarly to those based on additive effects, presumably as dominance variance can often be seen as additive variance under certain conditions (Hill et al., 2008). Overall, our findings suggest that integrating GWAS results into GS improves genomic GP models, enhances our understanding of candidate genes associated with resistance to VW, and increases the efficiency of the tomato breeding program.

## 5 CONCLUSIONS

The current study identified three wild tomato accessions (*S. peruvianum* LA2744, *S. arcanum* LA2157, and LA2172) as highly resistant to the *V. dahliae* race 3 strain KJ14a. Further evaluation is needed to assess these accessions in the field, to determine the potential of the newly identified QTL as a novel source of VW resistance in tomato breeding. Although these germplasm sources may carry undesirable horticultural traits, they remain excellent donors for backcrossing and marker‐assisted introgression. The primary strategy of combining LD‐based GWAS and GBLUP was to gain valuable insights into the genetic architecture of traits in our tomato panel. This information was then used to enhance the accuracy of the GBLUP model in predicting breeding values. By combining these approaches, we can make substantial progress in the genetic improvement of tomatoes. Additionally, the high predictive power of GWABLUP in our study demonstrated the potential for deploying GP, which plant breeders can effectively combine with donor alleles and desirable agronomic backgrounds. Looking ahead, converting candidate loci into breeder‐friendly markers, such as Kompetitive Allele‐Specific polymerase chain reaction (Semagn et al., 2014) or cleaved amplified polymorphic sequence (Thiel et al., 2004), and validating these markers in segregating populations will enhance marker‐assisted selection. By integrating GWABLUP with both phenotypic and genomic evaluations, we can accumulate minor QTLs and streamline the selection process for quantitative resistance to VW. Our holistic approach focuses on utilizing wild germplasm, developing and mapping new resistance loci, leveraging LD‐based GWAS and GP, and incorporating novel alleles into elite tomato lines. This strategy lays the foundation for a scalable framework for breeding long‐lasting resistance to *V. dahliae* race 3. By combining precise marker discovery with prediction models informed by GWAS, plant breeders can expedite the development of resistance to complex traits and improve sustainable disease management in tomato.

## AUTHOR CONTRIBUTIONS

**Tika B. Adhikar**i:Conceptualization; data curation; formal analysis; funding acquisition; investigation; methodology; project administration; writing—original draft; writing—review and editing. **Bode A. Olukolu**: Data curation; formal analysis; investigation; methodology; resources; software; validation; visualization; writing—original draft; writing—review and editing. **Anju Pandey**: Investigation; methodology; writing—review and editing. **Ashley N. Philbrick**: Investigation; methodology; writing—review and editing. **Dilip R. Panthee**: Funding acquisition; methodology; resources; writing—review and editing. **Reza Shekasteband**: Funding acquisition; resources; writing—review and editing. **Randolph G. Gardner**: Resources; writing—review and editing. **Ralph A. Dean**: Funding acquisition; resources; writing—review and editing. **Frank J. Louws**: Conceptualization; funding acquisition; methodology; project administration; writing—review and editing.

## CONFLICT OF INTEREST STATEMENT

The authors declare no conflicts of interest.

## Supporting information




**Fig. S1**. Boxplot showing read depth distribution across accessions (Y‐axis) after read depth filtering at a threshold of 6 across 235 tomato accessions.
**Fig. S2**. The proportion of genotypic classes in the tomato diversity population, including homozygotes (0/0 and 1/1; code as 0 and 2 for number of alternative alleles) and heterozygotes (0/1; code as 1 for number of alternative alleles). There are 42,941 markers or variants across 235 tomato accessions.
**Fig. S3**. A bar plot showing the read depth distribution across the tomato diversity panel and 42,941 markers across 235 tomato accessions. The median and mean read depth are shown for the population.
**Fig S4**. Plot showing minor allele frequency (MAF) distribution across 235 tomato accessions based on 42,941 markers. The median and mean MAF are shown for the population.
**Fig. S5**. Heat map showing missing rate with blue and red indicating called genotypes and missing genotypes, respectively. The heat map depicts 42,941 markers across 235 tomato accessions.
**Fig. S6**. The population structure analysis (top) conducted with the STRUCTURE software reveals three distinct subpopulations, along with an admixed group (A). The DeltaK plot (bottom left) identifies the optimal number of subpopulations (B). Additionally, the neighbor‐joining‐based phylogenetic tree, created with 10,000 bootstraps, is illustrated (C) at the bottom right. Both the structure and phylogenetic analyses are based on 8,578 markers and 226 tomato accessions.
**Fig S7**. Population structure analysis was conducted using principal component analysis (PCA) with 8,578 markers across 226 tomato accessions. The PCA clusters align with groupings identified through STRUCTURE software and phylogenetic analysis performed in DARwin software. The quality of each tomato accession's representation (cos2) is consistently high within each cluster. The most resistant accessions of *lycopersicum* (LA) are clustered together within the admixed group.
**Fig S8**. Population structure for various subpopulations (4‐7), as determined using 226 tomato accessions and 8,578 markers in the STRUCTURE software, consistently reveals 3 distinct subpopulations and an admixed group.
**Fig. S9**. Clustering analysis shows the number of subpopulations from 4 to 7 using 226 tomato accessions and 8,578 markers. The kinship matrix (or GRM: genetic relationship matrix) was used as input for generating the cladogram. The composition of the 3 subpopulations is mainly maintained.
**Fig. S10**. Manhattan plot (left panel) shows genome‐wide associations for resistance to *Verticillium dahliae* race 3 strain KJ14a using 8,578 markers or variants across 235 tomato accessions (**A**). The Y‐axis and X‐axis represent the ‐log10 of P‐values and chromosomal positions, respectively. The red and gray lines indicate the Bonferroni test and false discovery rate (FDR)‐based significance thresholds, respectively. Quantile‐Quantile (QQ) plots show observed versus expected *P*‐values (right panel) and reveal low FDR and high detection power (**B**).
**Fig. S11A‐D**. Quantile‐quantile (QQ) plots derived from genome‐wide association study (GWAS) results based on 8,578 markers with more stringent filtering for missing rate (variant missing rate = 20% and sample missing rate = 5%). False discovery is highly inflated when the GWAS model uses only Q (4 subpopulations) to control population structure. Using both Q and K sometimes results in a loss of detection power. Using only K typically produces the best result.
**Fig. S12A**. Local linkage disequilibrium (LD) between the trait‐associated marker (red text) and candidate genes identified on chromosomes 3, 5, and 7 during genome‐wide association analysis. LD‐based genome‐wide association analysis improves confidence in limiting association to a single defense‐related gene.
**Fig. S12B**. Local linkage disequilibrium (LD) between the trait‐associated marker and candidate genes identified on chromosome 10 during genome‐wide association analysis. LD‐based genome‐wide association analysis improves confidence in limiting association to a single defense‐related gene. Gene names in grey indicate that the trait‐associated marker is not in LD with the candidate gene, hence not declared as a candidate gene.

**File S1**. Excel file containing six spreadsheets: (1) phenotypic data; (2) genotypic data; (3) subpopulation membership of individuals from STRUCTURE analysis; (4) principal component analysis (PCA) results for the first five components; (5) candidate genes with associated metadata; and (6) the top 100 GWAS hits used for GWAS‐informed genomic prediction (GWABLUP).

## Data Availability

All genotypic data used in this study are available in Supporting information S1.
