# Supplementary material for: Genome‐wide association study identifies quantitative trait loci associated with resistance to Verticillium dahliae race 3 in tomato
Source: Plant Genome. 2025 Oct 14;18(4):e70132. doi: 10.1002/tpg2.70132 (PMC12521801; doi:10.1002/tpg2.70132)
Supplement: Supplementary file 1 — Fig. S1. Boxplot showing read depth distribution across accessions (Y‐axis) after read depth filtering at a threshold of 6 across 235 tomato accessions. Fig. S2. The proportion of genotypic classes in the tomato diversity population, including homozygotes (0/0 and 1/1; code as 0 and 2 for number of alternative alleles) and heterozygotes (0/1; code as 1 for number of alternative alleles). There are 42,941 markers or variants across 235 tomato accessions. Fig. S3. A bar plot showing the read depth distribution across the tomato diversity panel and 42,941 markers across 235 tomato accessions. The median and mean read depth are shown for the population. Fig S4. Plot showing minor allele frequency (MAF) distribution across 235 tomato accessions based on 42,941 markers. The median and mean MAF are shown for the population. Fig. S5. Heat map showing missing rate with blue and red indicating called genotypes and missing genotypes, respectively. The heat map depicts 42,941 markers across 235 tomato accessions. Fig. S6. The population structure analysis (top) conducted with the STRUCTURE software reveals three distinct subpopulations, along with an admixed group (A). The DeltaK plot (bottom left) identifies the optimal number of subpopulations (B). Additionally, the neighbor‐joining‐based phylogenetic tree, created with 10,000 bootstraps, is illustrated (C) at the bottom right. Both the structure and phylogenetic analyses are based on 8,578 markers and 226 tomato accessions. Fig S7. Population structure analysis was conducted using principal component analysis (PCA) with 8,578 markers across 226 tomato accessions. The PCA clusters align with groupings identified through STRUCTURE software and phylogenetic analysis performed in DARwin software. The quality of each tomato accession's representation (cos2) is consistently high within each cluster. The most resistant accessions of lycopersicum (LA) are clustered together within the admixed group. Fig S8. Population structure for [file TPG2-18-e70132-s001.pdf]

Supplementary Fig. S1

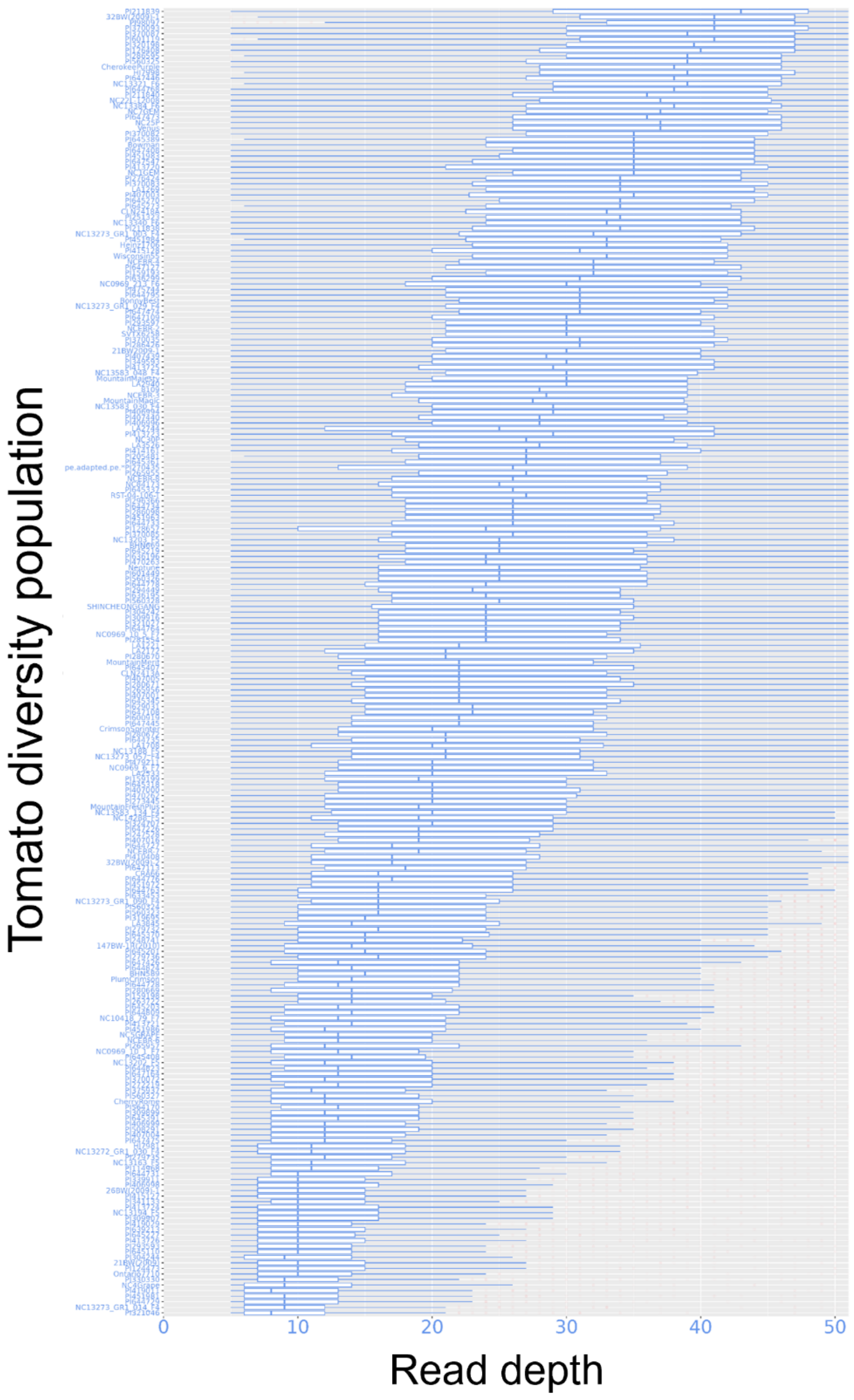

Supplementary Fig. S1. Boxplot showing read depth distribution across samples (Y-axis) after read depth filtering at a threshold of **6 across 235 tomato accessions.**

Supplementary Fig. S2

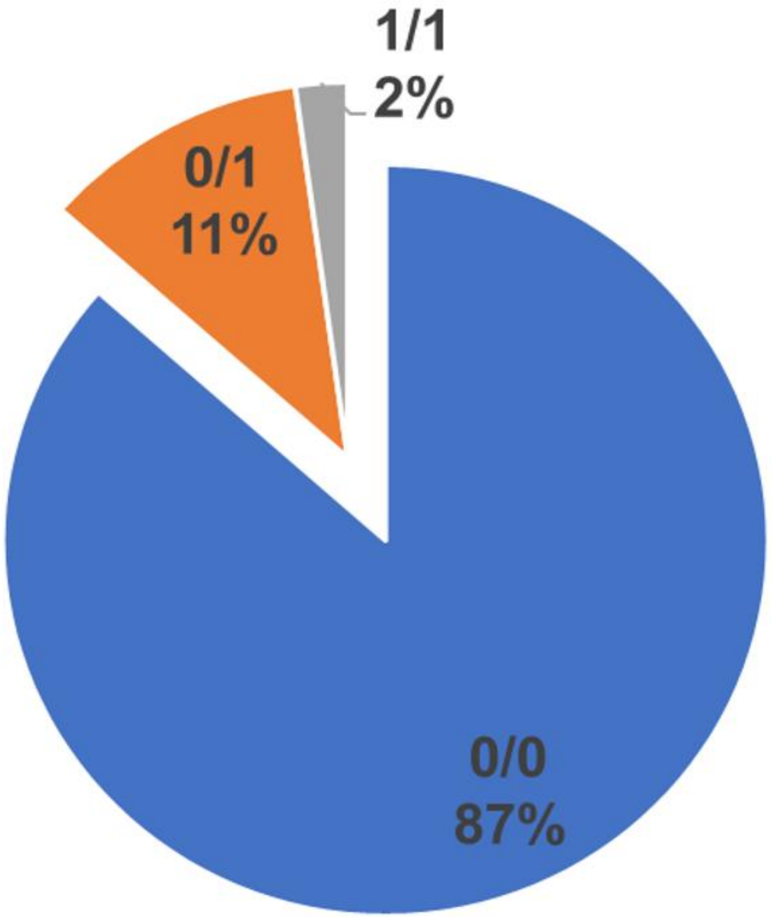

Supplementary Fig. S2. The proportion of genotypic classes in the tomato diversity population, including homozygotes (0/0 and 1/1; code as 0 and 2 for number of alternative alleles) and heterozygotes (0/1; code as 1 for number of alternative alleles). There are 42,941 markers or variants across 235 tomato accessions.

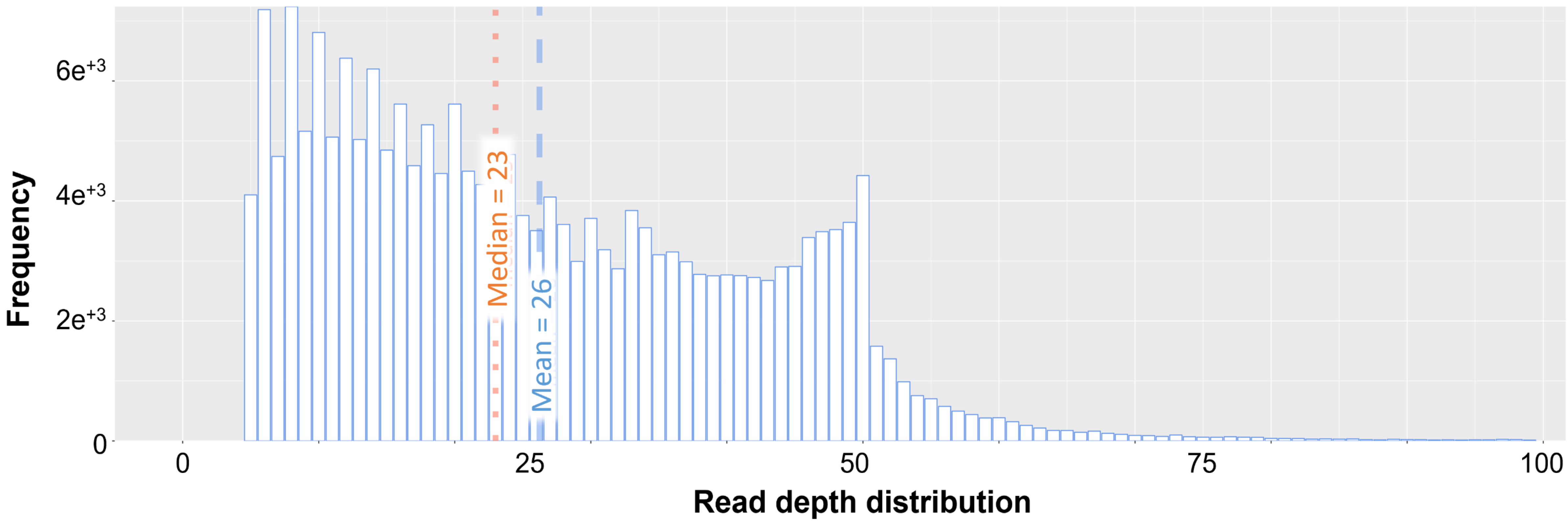

Supplementary Fig. S3. A bar plot showing the read depth distribution across the tomato diversity panel and 42,941 markers across 235 tomato accessions. The median and mean read depth are shown for the population.

Supplementary Fig. S4

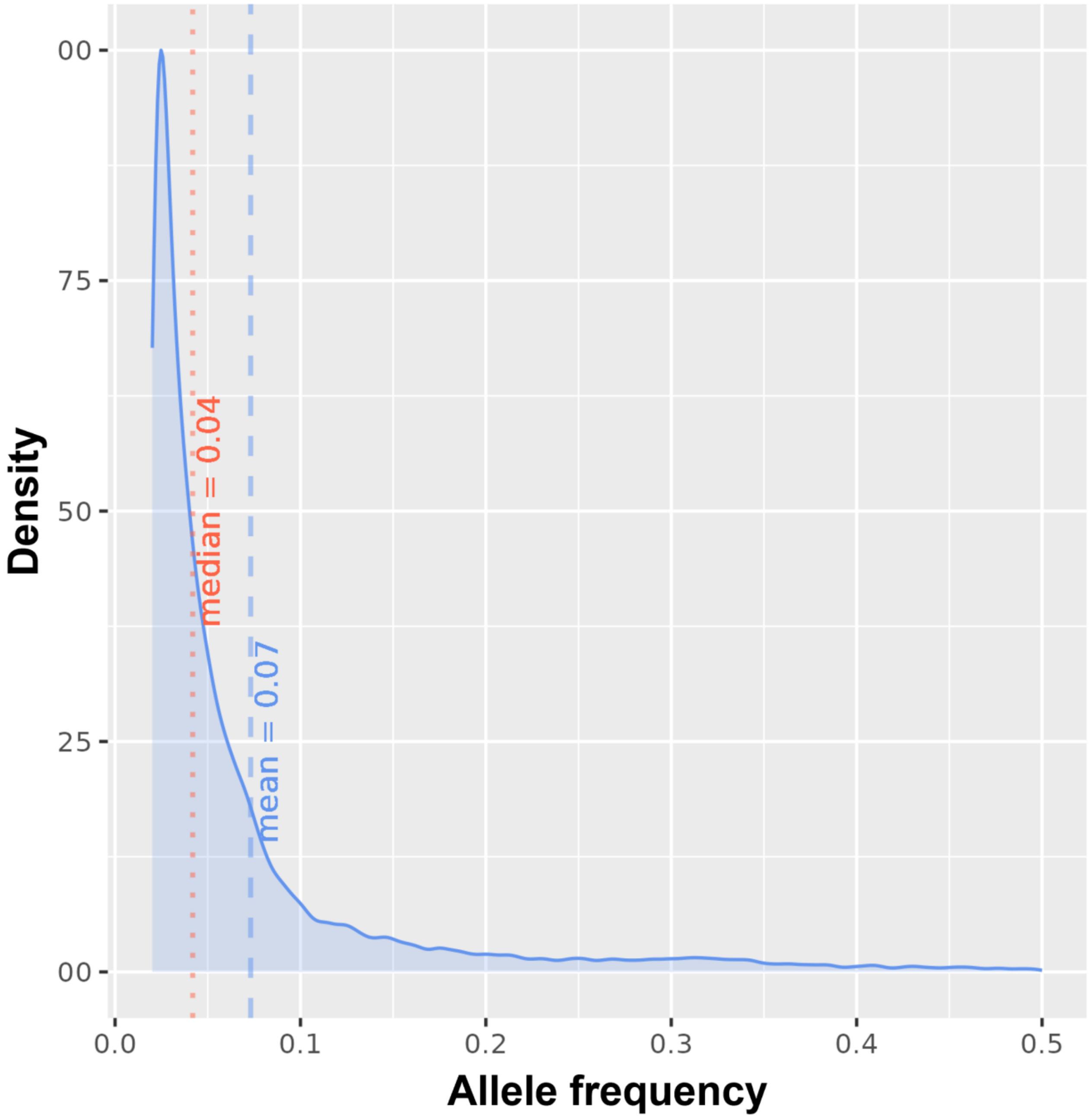

Supplementary Fig S4. Plot showing minor allele frequency (MAF) distribution across 235 tomato accessions based on 42,941 markers. The median and mean MAF are shown for the population.

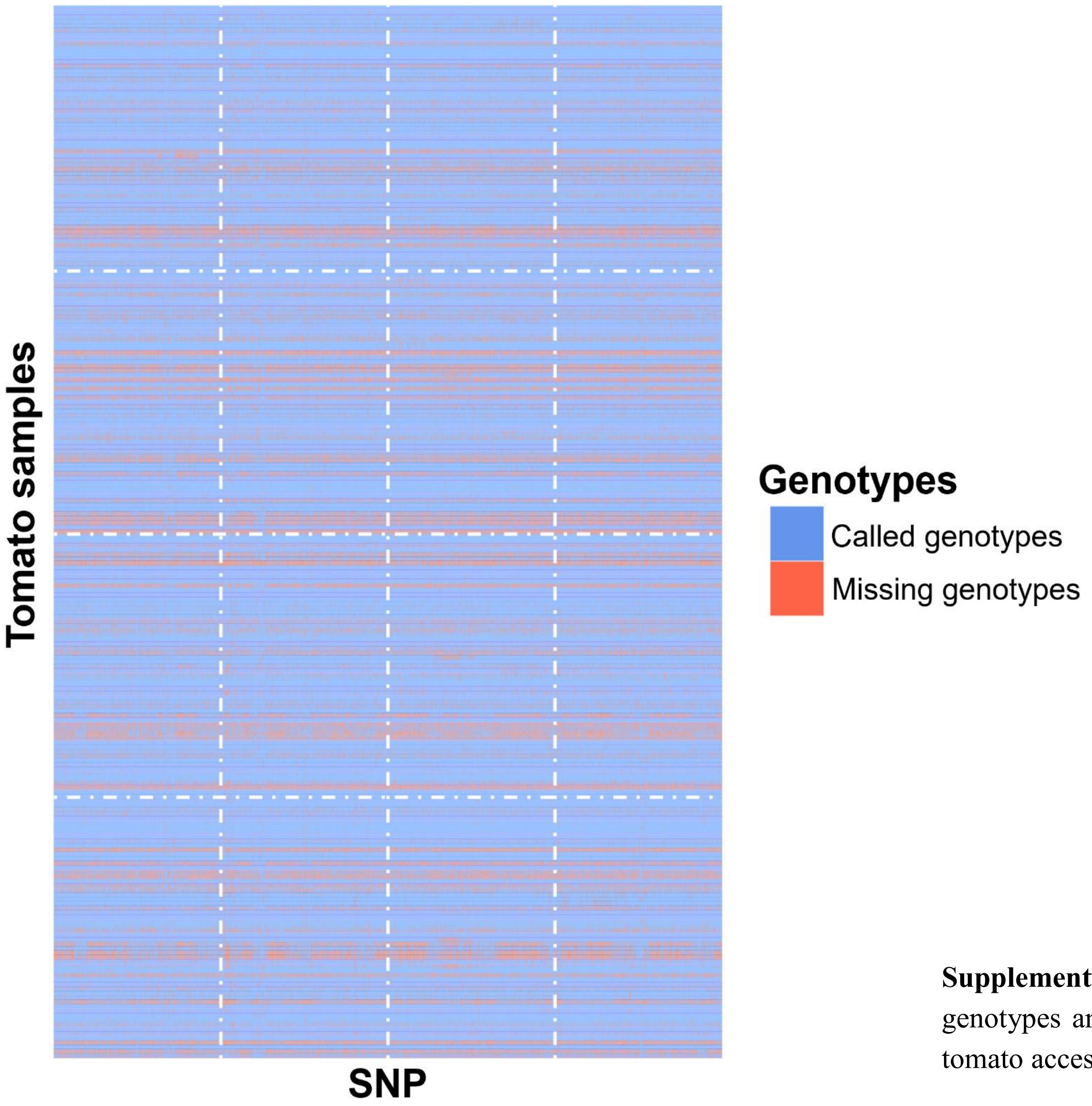

Supplementary Fig. S5. Heat map showing missing rate with blue and red indicating called genotypes and missing genotypes, respectively. The heat map depicts 42,941 markers across 235 tomato accessions.

Fig. S6

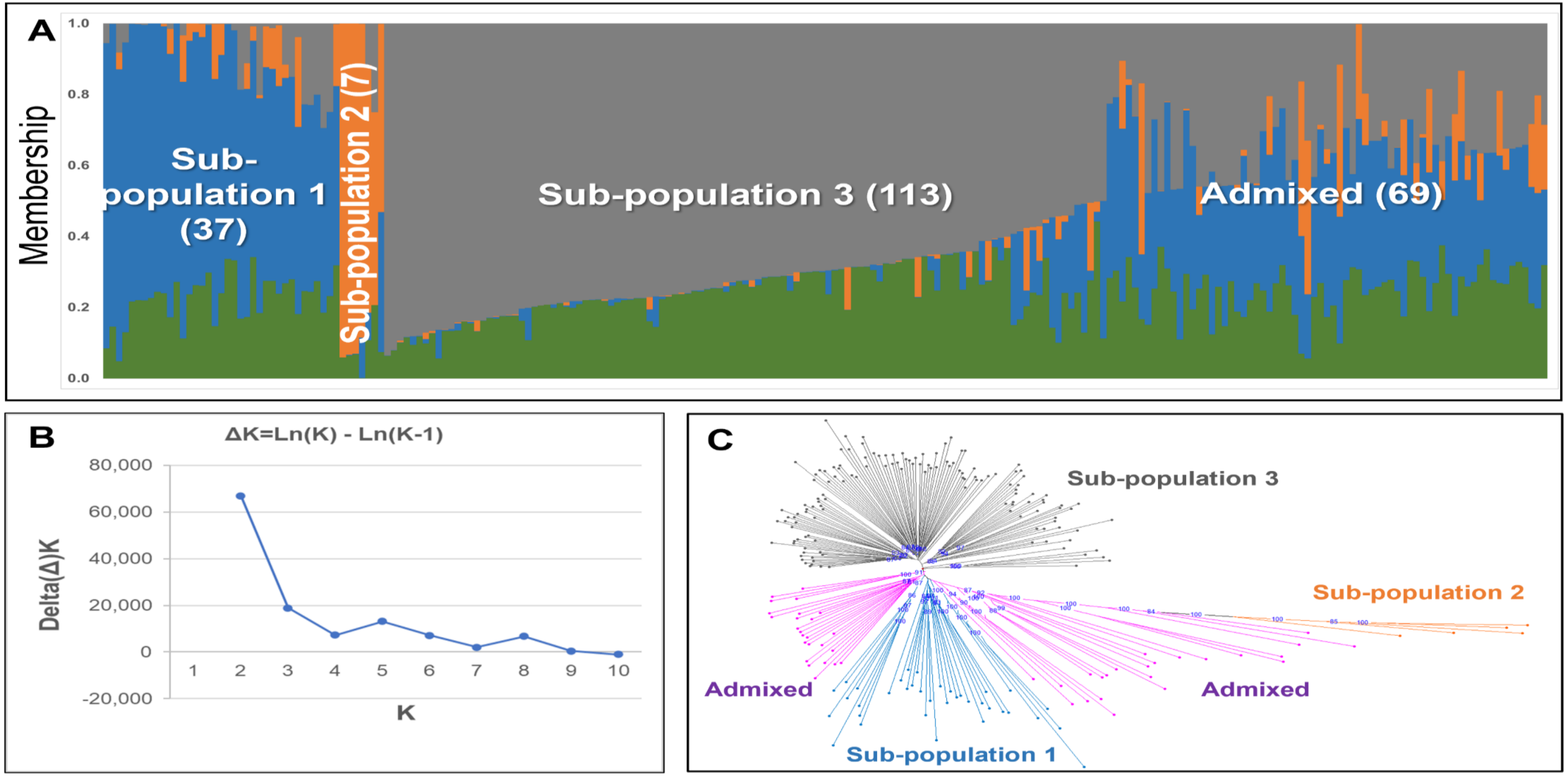

Fig. S6. The population structure analysis (top) conducted with the STRUCTURE software reveals three distinct subpopulations, along with an admixed group (**A**). The DeltaK plot (bottom left) identifies the optimal number of subpopulations (**B**). Additionally, the neighbor-joining-based phylogenetic tree, created with 10,000 bootstraps, is illustrated (C) at the bottom right. Both the structure and phylogenetic analyses are based on 8,578 markers and 226 tomato accessions.

Fig. S7

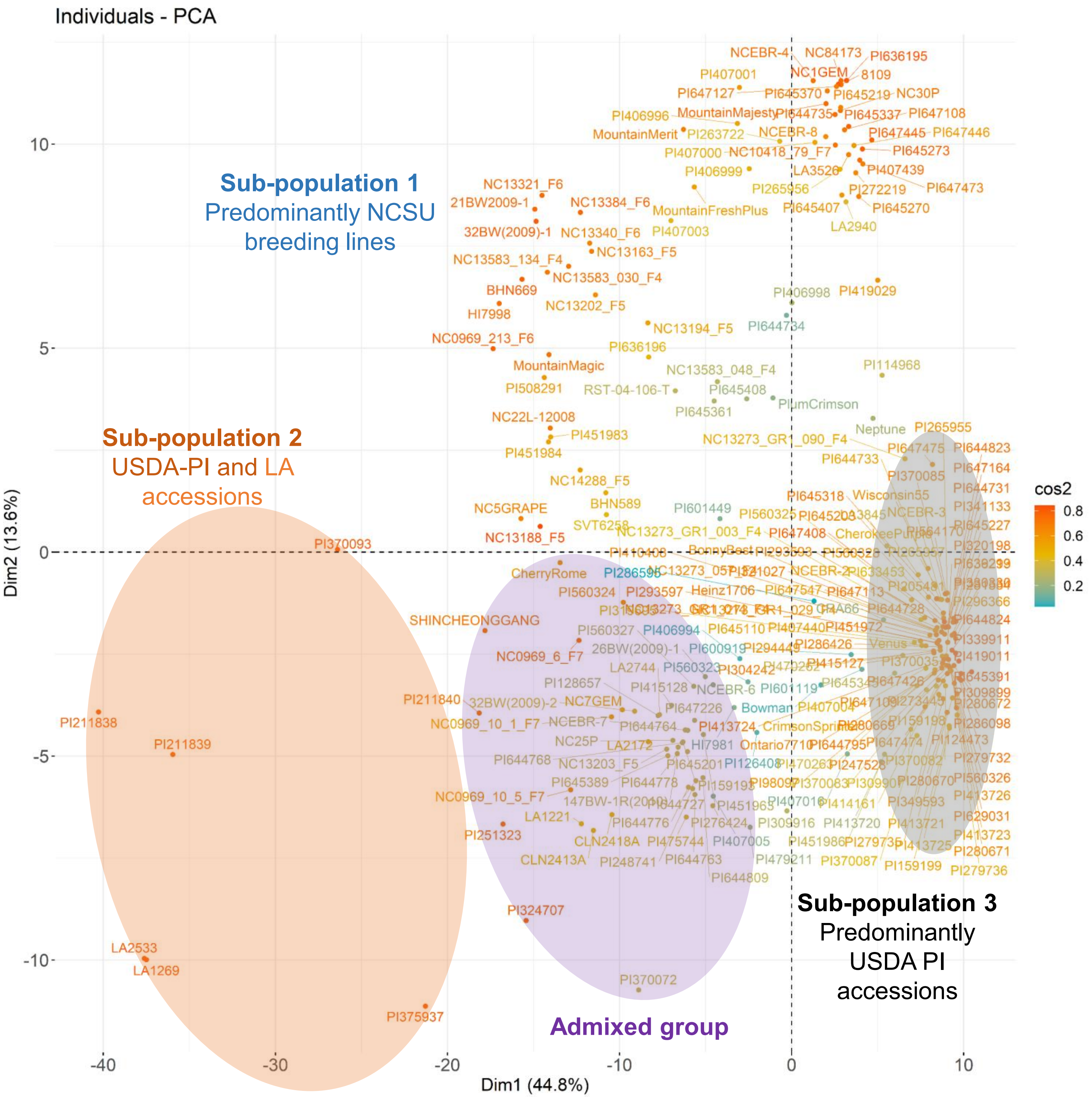

Fig S7. Population structure analysis was conducted using principal component analysis (PCA) with 8,578 markers across 226 tomato accessions. The PCA clusters align with groupings identified through STRUCTURE software and phylogenetic analysis performed in DARwin software. The quality of each tomato genotype's representation (cos2) is consistently high within each cluster. Resistant accessions of *lycopersicum* (LA) are clustered together within the admixed group.

Supplementary Fig. S8

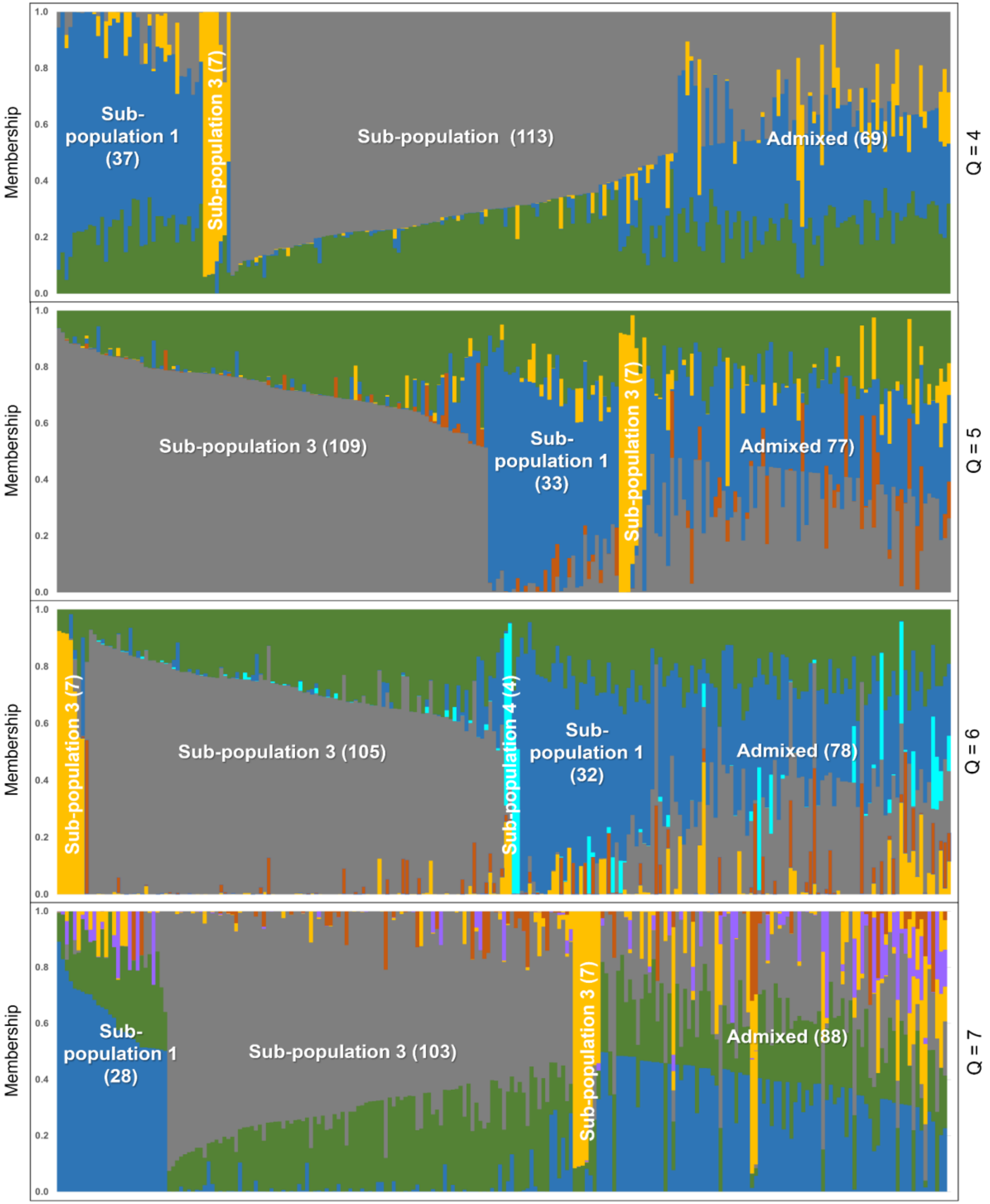

Supplementary Fig S8. Population structure for various subpopulations (4-7), as determined using 226 tomato accessions and 8,578 markers in the STRUCTURE software, consistently reveals 3 distinct subpopulations and an admixed group.

Supplementary Fig. S9

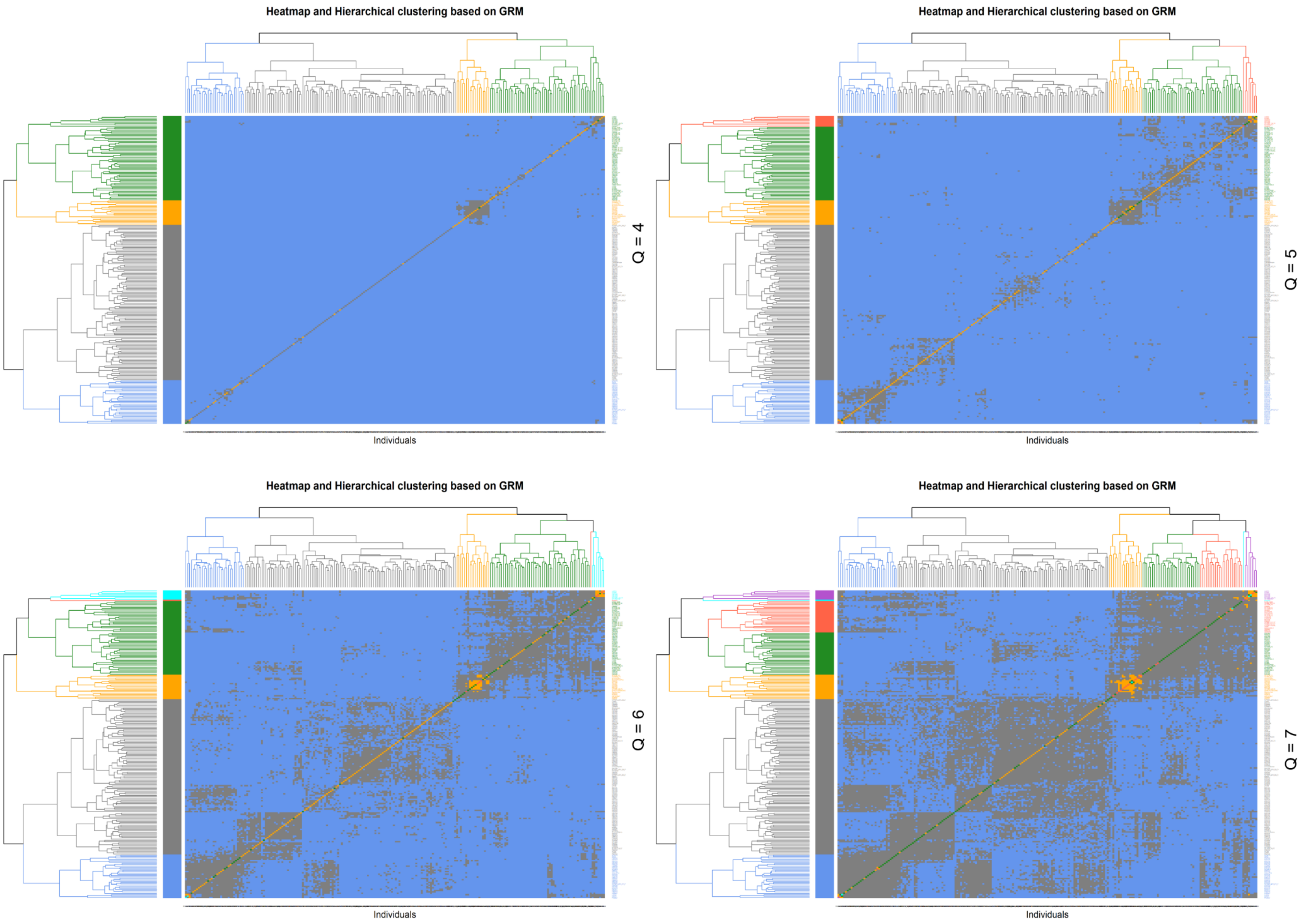

Supplementary Fig. S9. Clustering analysis shows the number of subpopulations from 4 to 7 using 226 tomato accessions and 8,578 markers. The kinship matrix (or GRM: genetic relationship matrix) was used as input for generating the cladogram. The composition of the 3 subpopulations is mainly maintained.

Supplementary Fig. S10

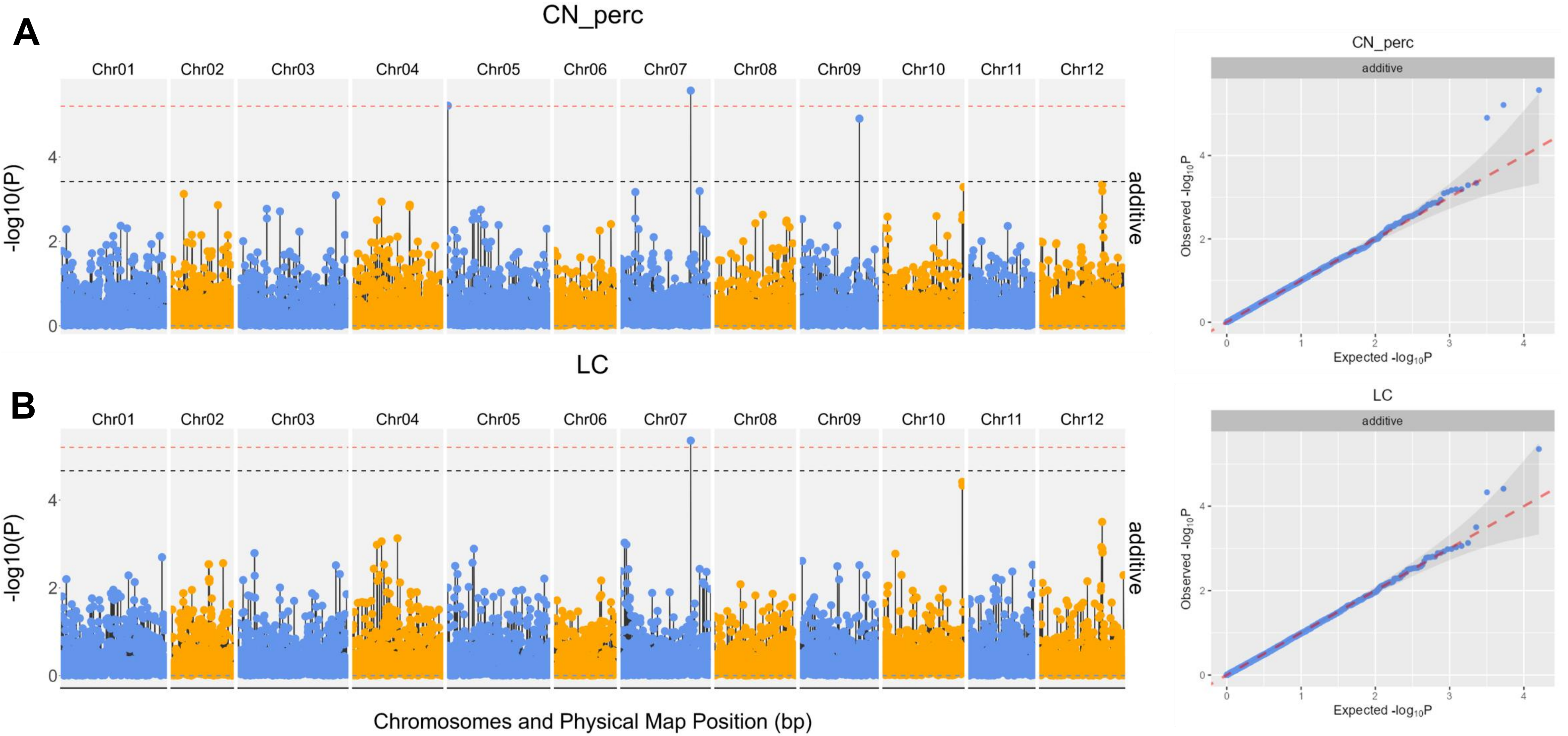

Supplementary Fig. S10. Manhattan plot (left panel) shows genome-wide associations for resistance to *Verticillium dahliae* race 3 strain KJ14a using **8,578 markers or variants across 205 tomato accessions** (**A**). Y-axis and X-axis show -log10 of *P*-values and the chromosomal positions, respectively. The red and gray lines indicate the Bonferroni test and false discovery rate (FDR)-based significance thresholds, respectively. Quantile-Quantile (QQ) plots show observed versus expected *P*-values (right panel) and reveal low FDR and high detection power (**B**).

Supplementary Fig. S11A

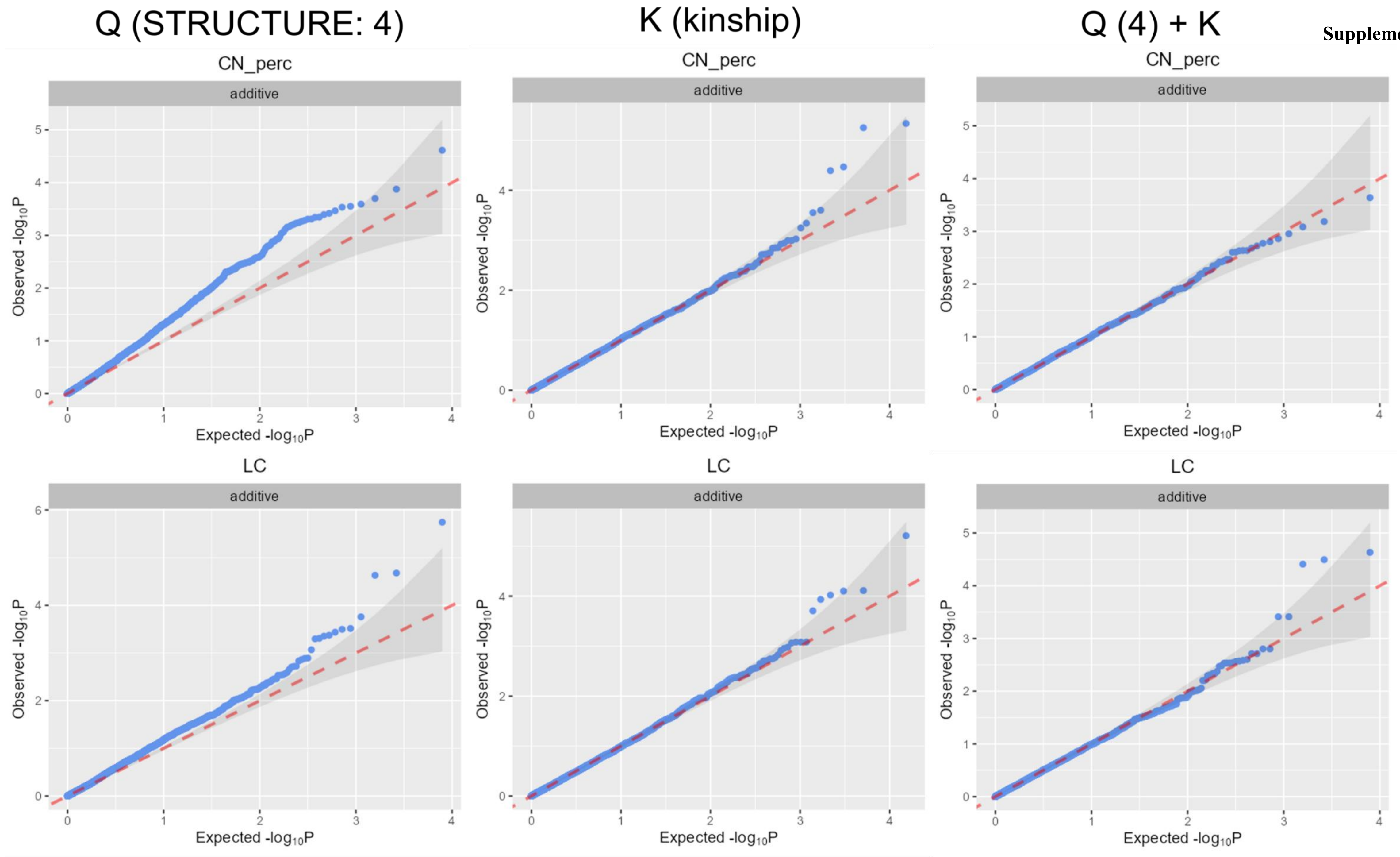

Supplementary Fig. S11B

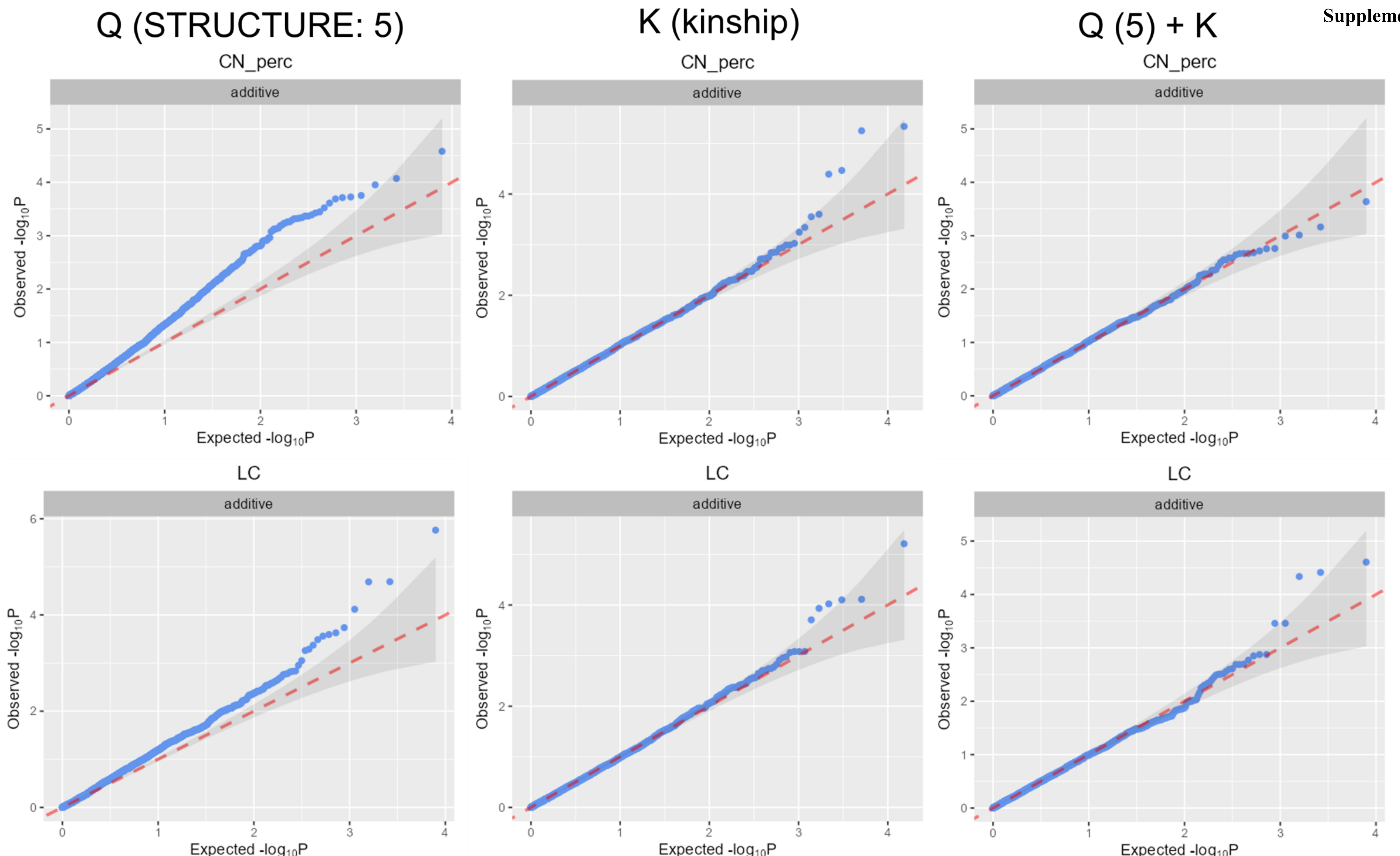

Supplementary Fig. S11C

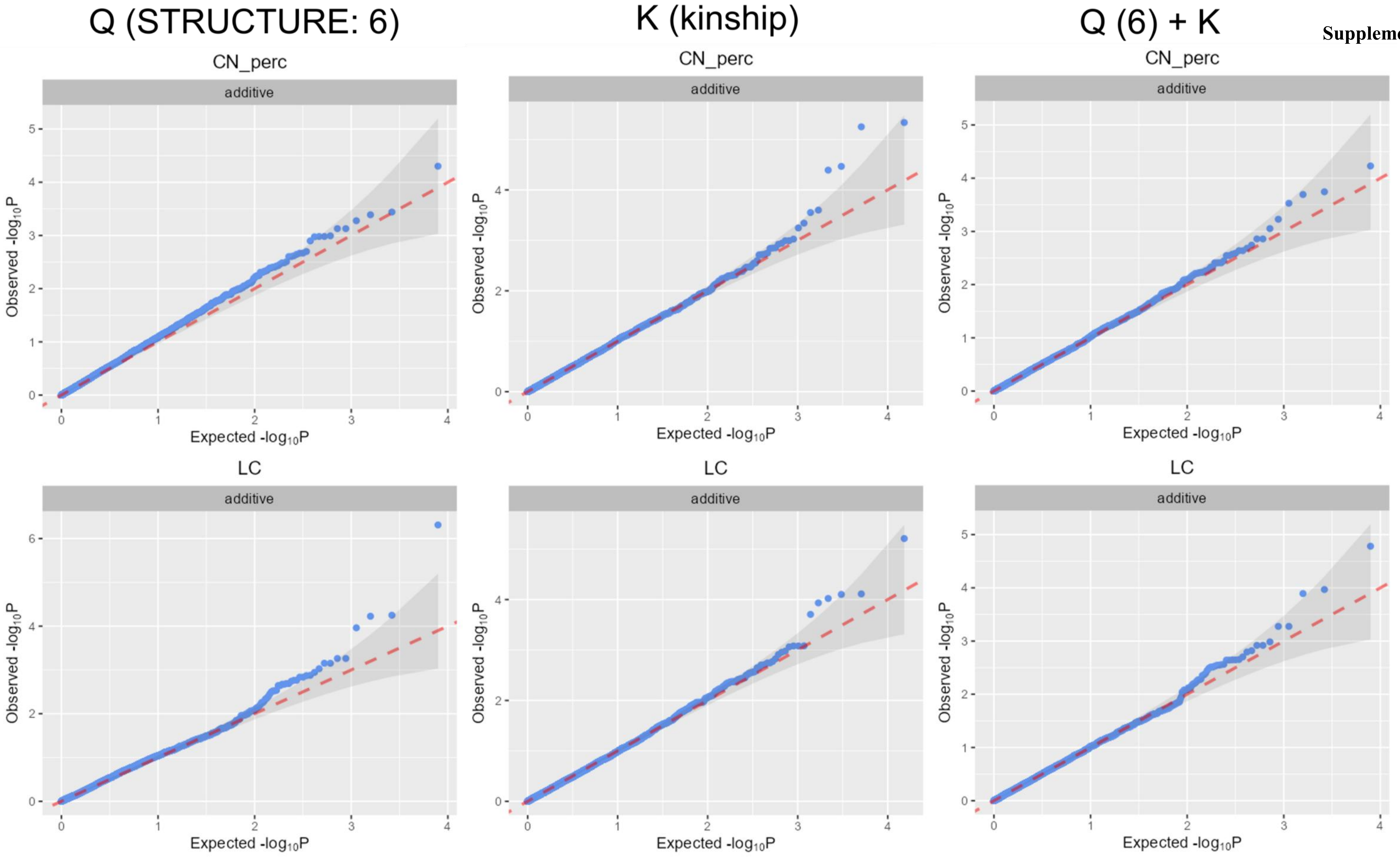

Supplementary Fig. 11D

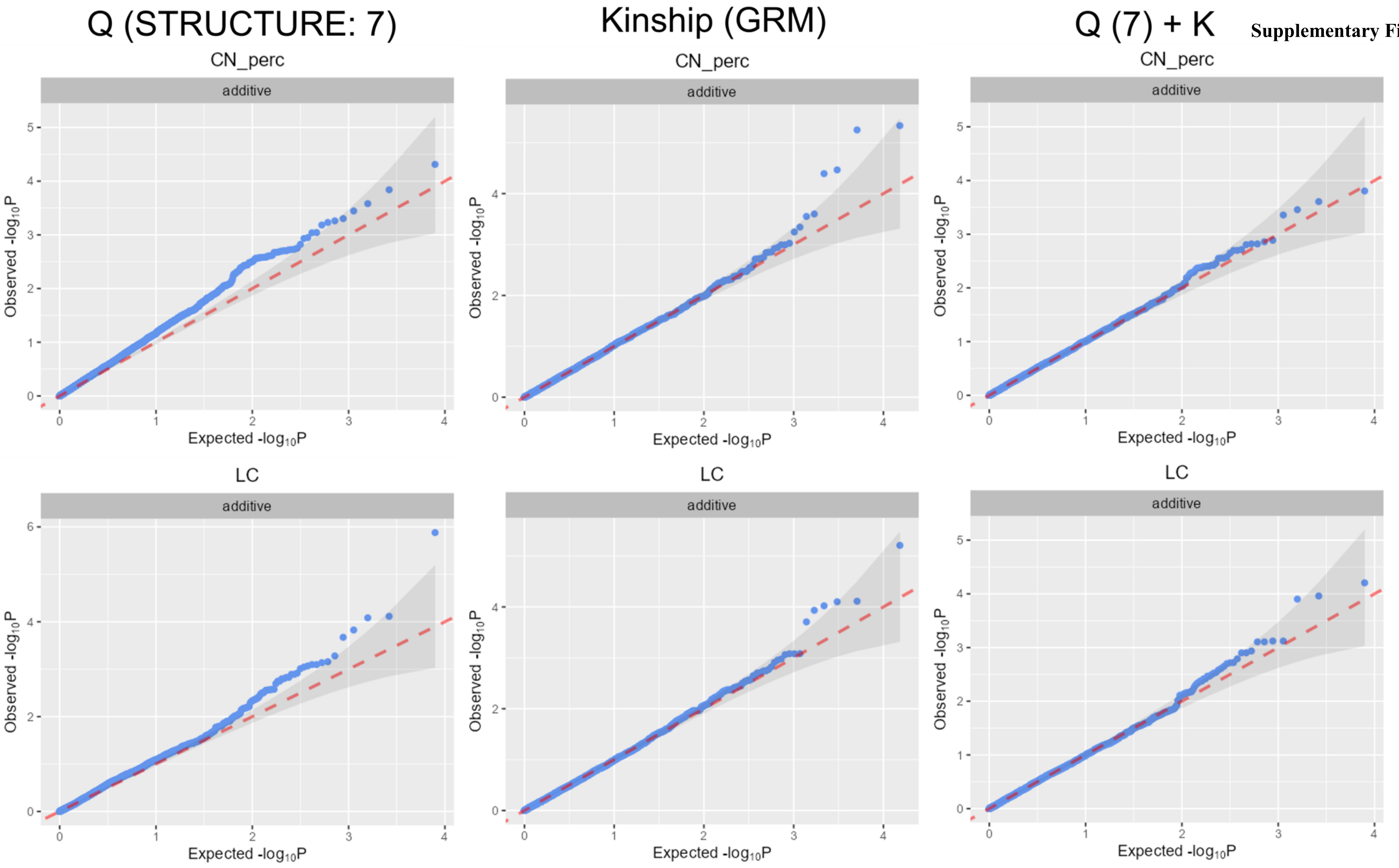

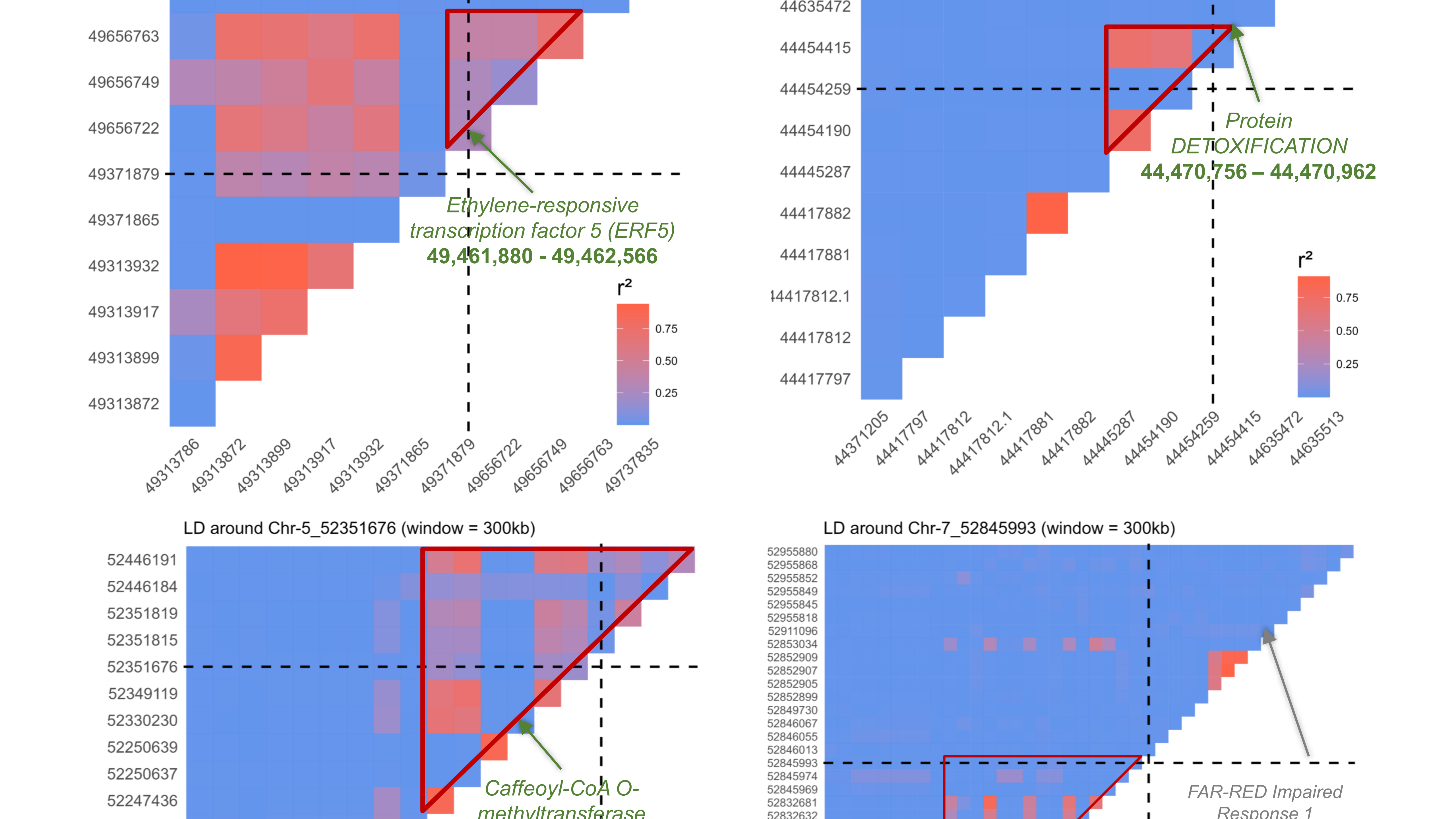

49656763
49656749
49656722
49371879
49371865
49313932
49313917
49313899
49313872
49313786
49313872
49313899
49313917
49313932
49371865
49371879
49656722
49656749
49656763
49737835
Ethylene-responsive transcription factor 5 (ERF5)
49,461,880 - 49,462,566
r²
0.75
0.50
0.25
44635472
44454415
44454259
44454190
44445287
44417882
44417881
44417812.1
44417812
44417797
44371205
44417797
44417812
44417812.1
44417881
44417882
44445287
44454190
44454259
44454415
44635472
44635513
Protein DETOXIFICATION
44,470,756 – 44,470,962
r²
0.75
0.50
0.25
LD around Chr-5_52351676 (window = 300kb)
52446191
52446184
52351819
52351815
52351676
52349119
52330230
52250639
52250637
52247436
Caffeoyl-CoA O-methyltransferase
LD around Chr-7_52845993 (window = 300kb)
52955880
52955868
52955852
52955849
52955845
52955818
52911096
52853034
52852909
52852907
52852905
52852899
52849730
52846067
52846055
52846013
52845993
52845974
52845969
52832681
52832632
FAR-RED Impaired Response 1

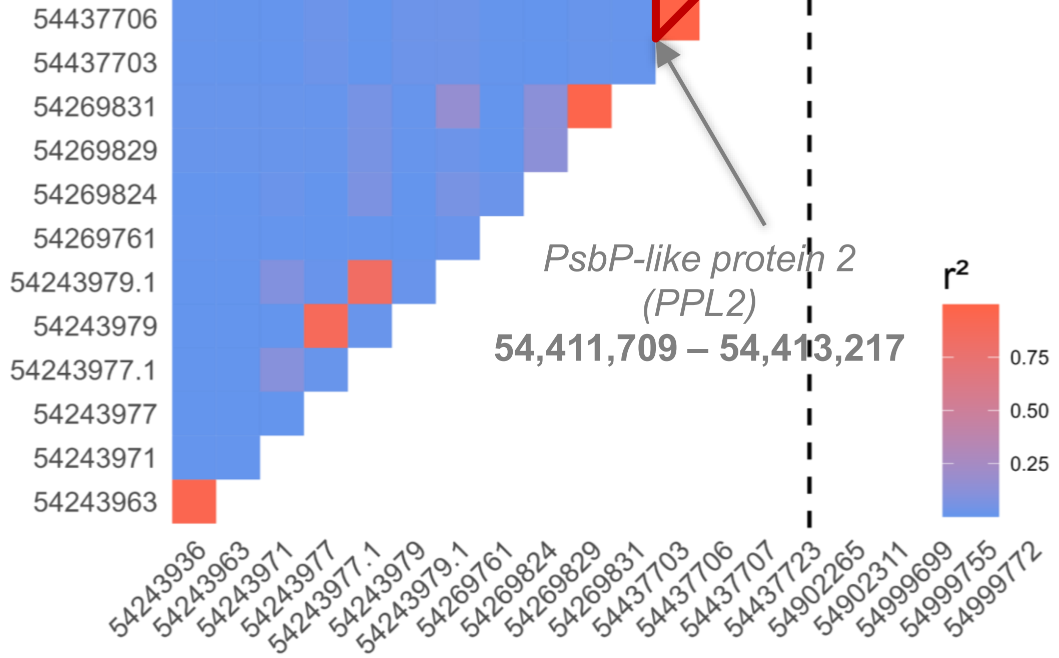

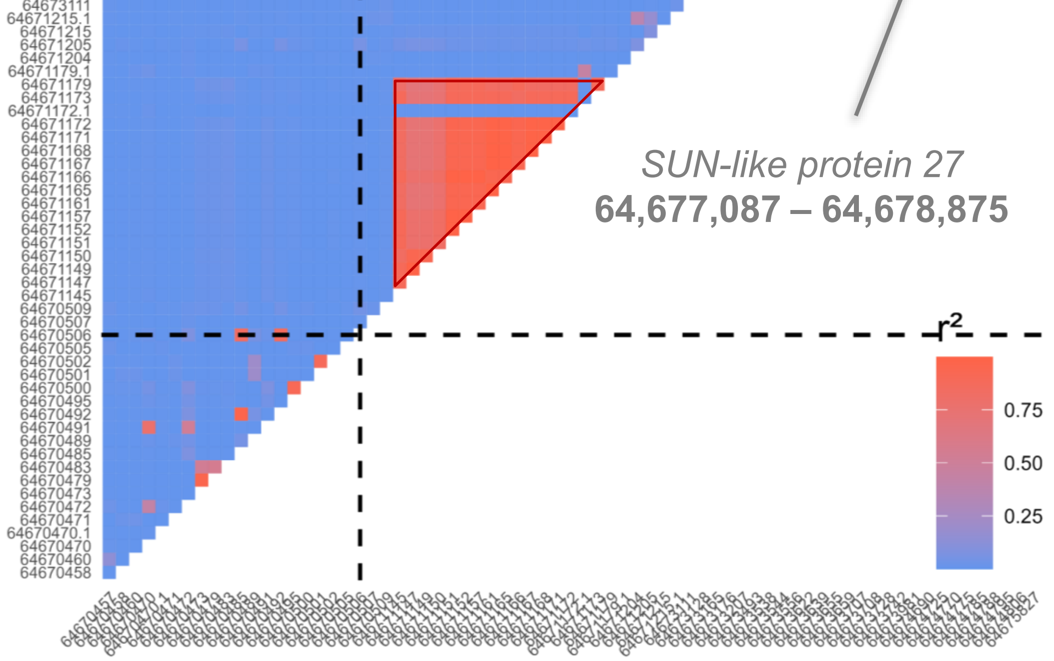

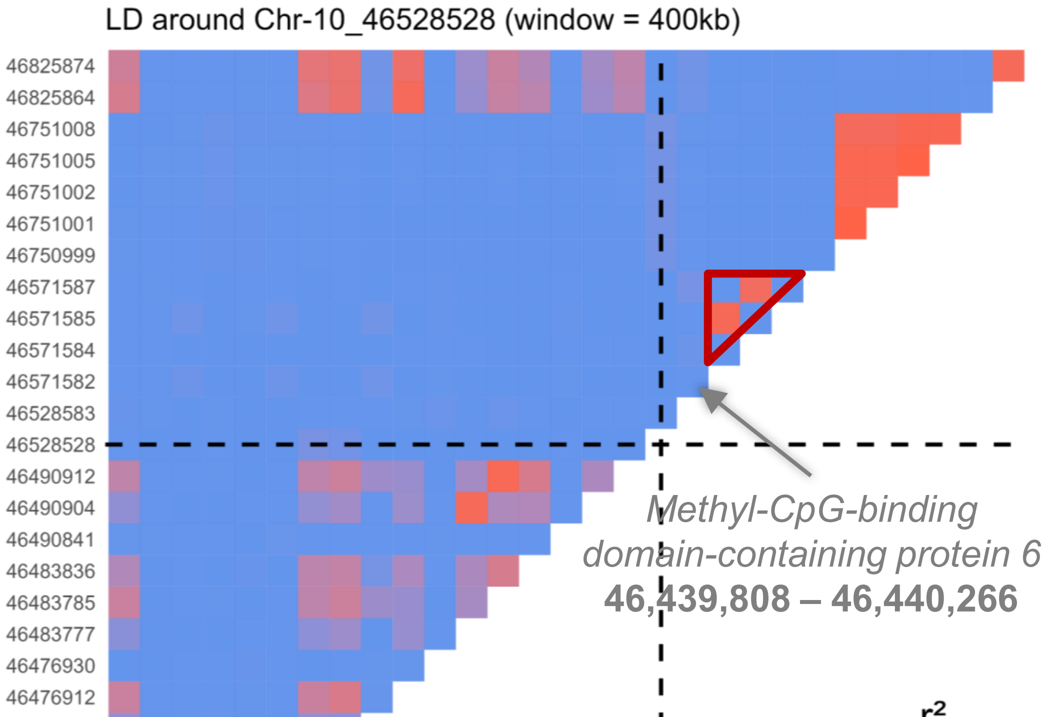

Supplementary Fig. S12B. Local linkage disequilibrium (LD) between the trait-associated marker (red text) and candidate genes identified on **chromosome 10** during genome-wide association analysis. LD-based genome-wide association analysis improves confidence in limiting association to a single defense-related gene. Gene names in grey indicate that the trait-associated marker is not in LD with the candidate gene, hence not declared as a candidate gene.
